# A Subset of Latency-Reversing Agents Expose HIV-Infected Resting CD4^+^ T-Cells to Recognition by Cytotoxic T-Lymphocytes

**DOI:** 10.1371/journal.ppat.1005545

**Published:** 2016-04-15

**Authors:** R. Brad Jones, Stefanie Mueller, Rachel O’Connor, Katherine Rimpel, Derek D. Sloan, Dan Karel, Hing C. Wong, Emily K. Jeng, Allison S. Thomas, James B. Whitney, So-Yon Lim, Colin Kovacs, Erika Benko, Sara Karandish, Szu-Han Huang, Maria J. Buzon, Mathias Lichterfeld, Alivelu Irrinki, Jeffrey P. Murry, Angela Tsai, Helen Yu, Romas Geleziunas, Alicja Trocha, Mario A. Ostrowski, Darrell J. Irvine, Bruce D. Walker

**Affiliations:** 1 The Ragon Institute of Massachusetts General Hospital, Massachusetts Institute of Technology, and Harvard University, Cambridge, Massachusetts, United States of America; 2 Koch Institute for Integrative Cancer Research, MIT, Cambridge, Massachusetts, United States of America; 3 Department of Microbiology Immunology and Tropical Medicine, The George Washington University, Washington, D.C., United States of America; 4 Gilead Sciences, Foster City, California, United States of America; 5 Altor BioScience Corporation, Miramar, Florida, United States of America; 6 Center for Virology and Vaccine Research, Beth Israel Deaconess Medical Center, Harvard Medical School, Boston, Massachusetts, United States of America; 7 The Maple Leaf Medical Clinic, Toronto, Ontario, Canada; 8 Department of Medicine, University of Toronto, Toronto, Ontario, Canada; 9 Li Ka Shing Medical Institute, St. Michael’s Hospital, Toronto, Ontario, Canad; 10 Howard Hughes Medical Institute, Chevy Chase, Maryland, United States of America; 11 Department of Biological Engineering, MIT, Cambridge, Massachusetts, United States of America; University of Wisconsin, UNITED STATES

## Abstract

Resting CD4^+^ T-cells harboring inducible HIV proviruses are a critical reservoir in antiretroviral therapy (ART)-treated subjects. These cells express little to no viral protein, and thus neither die by viral cytopathic effects, nor are efficiently cleared by immune effectors. Elimination of this reservoir is theoretically possible by combining latency-reversing agents (LRAs) with immune effectors, such as CD8^+^ T-cells. However, the relative efficacy of different LRAs in sensitizing latently-infected cells for recognition by HIV-specific CD8^+^ T-cells has not been determined. To address this, we developed an assay that utilizes HIV-specific CD8^+^ T-cell clones as biosensors for HIV antigen expression. By testing multiple CD8^+^ T-cell clones against a primary cell model of HIV latency, we identified several single agents that primed latently-infected cells for CD8^+^ T-cell recognition, including IL-2, IL-15, two IL-15 superagonists (IL-15SA and ALT-803), prostratin, and the TLR-2 ligand Pam_3_CSK_4_. In contrast, we did not observe CD8^+^ T-cell recognition of target cells following treatment with histone deacetylase inhibitors or with hexamethylene bisacetamide (HMBA). In further experiments we demonstrate that a clinically achievable concentration of the IL-15 superagonist ‘ALT-803’, an agent presently in clinical trials for solid and hematological tumors, primes the natural *ex vivo* reservoir for CD8^+^ T-cell recognition. Thus, our results establish a novel experimental approach for comparative evaluation of LRAs, and highlight ALT-803 as an LRA with the potential to synergize with CD8^+^ T-cells in HIV eradication strategies.

## Author Summary

Although modern therapies have greatly improved the lives of HIV-positive people with access to care, a cure remains elusive. This leaves these individuals burdened by a lifelong commitment to medication, and fails to fully restore health. Curing infection would likely require therapies that combine the ability to force the virus out the ‘latent state’ in which it hides, with immune responses able to kill unmasked infected cells, the so called “shock and kill” strategy. A critical aspect of this strategy is identifying drugs that are effective at shocking virus out of latency, known as latency reversing agents. In this study, we took the novel approach of using CD8^+^ T-cells, immune cells responsible for killing infected cells, as biosensors able to detect the unmasking of latently-infected cells. Using this method, we screened a panel of potential latency reversing agents. We found that while a subset of these agents exposed infected cells to the immune system, others did not. Our results establish a new method for screening potential latency reversing agents, and support the prioritization of the agents that were shown to be effective for combination with CD8^+^ T-cells in shock and kill strategies aimed at curing HIV infection.

## Introduction

Current antiretroviral (ARV) treatment regimens effectively suppress HIV replication, but are unable to cure infection. Viral persistence in long term cellular reservoirs leaves even well-treated individuals with a lifelong commitment to drug regimens, burdened by co-morbidities such as cardiovascular disease and neurocognitive disorders, and exposed to the negative social issues that come with being HIV-positive[1–3]. The development of therapeutic strategies capable of eradicating virus from individuals would greatly improve the lives of people living with HIV/AIDS.

Achieving viral eradication will be a complex task, involving the elimination or inactivation of virus that persists in multiple reservoirs, particularly in resting CD4^+^ T-cells, a major reservoir that will need to be addressed as part of any curative strategy. While in a quiescent state, HIV-infected resting CD4^+^ T-cells do not spontaneously produce virions and express little or no HIV antigen, and thus are neither killed by viral cytopathic effects, nor effectively targeted by immune effectors[4–7]. Rather, they persist as a stable reservoir that decays with a half-life of 44 months in ARV-treated individuals [8,9], and which can re-seed systemic infection upon ARV interruption. The “shock-and-kill” paradigm proposes to combine a latency-reversing agent (LRA) with immune effectors, such as CD8^+^ cytotoxic T-lymphocytes or NK cells, to selectively eliminate HIV-infected resting CD4+ T-cells[10].

The discovery and validation of LRAs has been approached using a number of different models of latency, and with diverse methods of assessing viral reactivation, leading to some debate over the effectiveness of many of these compounds[11]. The most prominent class of LRAs under exploration is the histone deacetylase inhibitors (HDAC inhibitors), which include SAHA (suberoylanilide hydroxamic acid or vorinostat), romidepsin, and panobinostat. While each of these HDAC inhibitors clearly induce the production of both viral RNA and protein from a number of cell line models of HIV latency, including ACH2 cells[12,13], their impact on latency in primary human cell models is less clear. For example, while some studies have demonstrated that SAHA induces the expression of viral proteins (or reporter genes) in primary cell models[12,14–17], others have observed the induction of viral RNA without detectable translation[15]. Similarly, while all three HDAC inhibitors have been shown to increase levels of HIV transcripts in *ex vivo* patient samples, the majority of studies have reported a lack of detectable virion production following treatment with SAHA and panobinostat, though virion production is induced at low levels by romidepsin[11,17–20]. The disconnect between HIV transcription and translation may be at least partially attributable to the production of ‘readthrough transcripts’ comprising host genes with integrated HIV sequence that is not bound for translation[19]. These complexities have implications for the interpretation of clinical trials. For example, whereas the administration of SAHA to ARV-treated HIV-infected subjects resulted in increased levels of cell-associated HIV transcripts[21,22], it is not clear that this was indicative of the induction of the HIV antigen expression needed for immune targeting of infected cells for elimination.

While induction of cell-associated HIV RNA may be insufficient for an effective LRA, requiring an LRA to induce viral particle production may be in excess of what is needed, in particular for CD8^+^ T-cell-based shock-and-kill approaches. T-cells can detect even a single MHC-peptide complex on a cell surface[23], and thus T-cells should be able to eliminate targets that translate very small amounts of HIV antigens, not necessarily associated with cellular activation or the production of detectable virions. The most clinically desirable LRAs may fall below this latter threshold, both because LRAs that induce virion production tend to be associated with high levels of bystander T-cell activation[19], and because the induced release of infectious virions may not be ideal, even under the cover of ART.

In this study, we present a novel approach utilizing HIV-specific CD8^+^ T-cell clones as biosensors to detect HIV latency reversal, thus incorporating a threshold of detection that is intrinsically relevant to CD8^+^ T-cell-based shock-and-kill eradication strategies. This is analogous to the commonplace use of monoclonal antibodies to detect proteins, with the additional advantage that, upon detecting intracellular antigen expression, CD8^+^ T-cells amplify this signal by the production of substantial amounts of cytokines, which can in turn be detected by ELISA. Using this method, we identify IL-15, two IL-15 superagonists (IL-15SA–generating by mixing recombinant IL-15 and IL-15Rα-FC and ALT-803 –a compound comprising a human IL-15N72D mutein bound to the human IL-15RαSu/Fc–see Discussion), IL-2, prostratin and Pam_3_CSK_4_ as LRAs that prime latently-infected targets for recognition by CD8^+^ T-cells. We further show that ALT-803, which is currently in a number of clinical trials for solid tumors and haematological malignancies, directly enhances the abilities of HIV-specific CD8^+^ T-cell clones and *ex vivo* bulk CD8^+^ T-cells to eliminate HIV-infected cells.

## Results

### HIV-specific CD8^+^ T-cells do not exhibit detectable recognition of latently-infected resting CD4^+^ T-cells in a primary cell model of latency

Although it is generally assumed that the induction of HIV antigen expression by an LRA will be needed to facilitate immunological clearance of infected resting CD4^+^ T-cells[10], this has not been formally tested. HIV transcripts, including processive polyadenylated HIV mRNAs are detectable in *ex vivo* purified resting CD4^+^ T-cells from ARV-treated patients[24]. Given the high sensitivity of CD8^+^ T-cells, it is plausible that sufficient antigen expression may occur in a latently-infected cell to trigger an immune response. It has also been suggested that the expression of particular HIV antigens, such as Gag, may persist even in an otherwise latent state[25,26].

**Fig 1:**
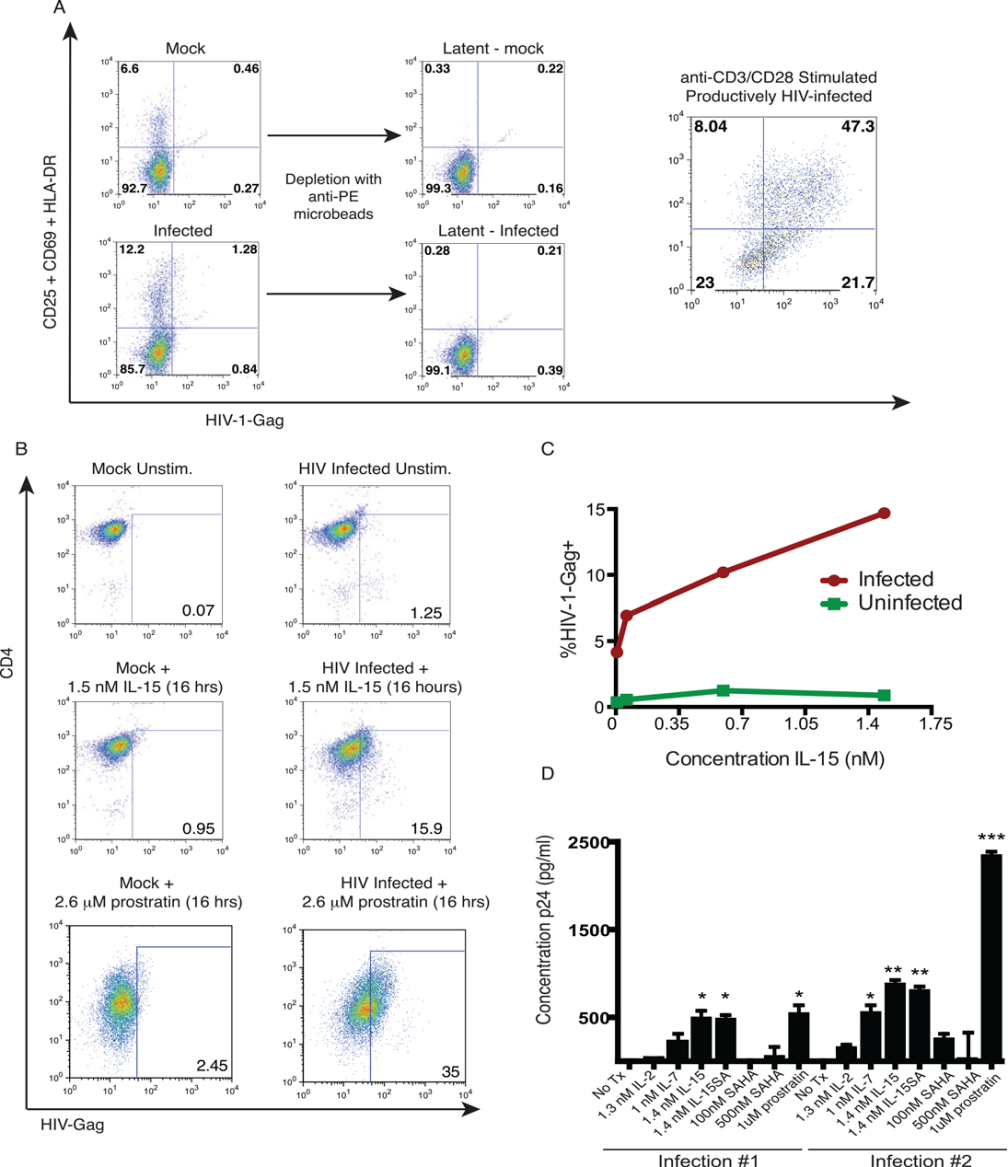
Induction of HIV by LRAs in a primary CD4+ T-cell direct infection latency model. CD4^+^ T-cells were isolated from PBMC by negative selection, cultured with CCL19 and then magnetofected with HIV, or mock infected (no virus). Two days later, cells were depleted of activated cells. We defined activated cells as those expressing at least one of CD69, HLA-DR, or CD25 based on a study that defined CD4+ T-cells with the triple-negative phenotype as quiescent[29]. In parallel, CD4^+^ T-cells were activated using anti-CD3/anti-CD28 antibodies and infected with HIV (productively infected). **A**. Depletion of activated cells by anti-PE microbeads. Shown are flow cytometry data gated on CD3^+^CD4^+^ lymphocytes (95% pure) and depicting CD25/CD69/HLA-DR staining (all pooled on PE channel)–y-axis, by HIV-Gag–x-axis. The left and middle panels represent pre- and post-depletion of activated cells, respectively to generate latently-infected cells. The right panel shows productively-infected target cells. **B**. Latently infected or mock-infected resting CD4^+^ T-cells were prepared as in **A** and then either stimulated with 1.5 nM IL-15, with 2.6 μM prostratin, or left unstimulated for 36 hours. Shown are flow cytometry data, gated on lymphocytes (SSC/FSC) and depicting CD4 –y-axis, by HIV-Gag–x-axis. **C**. In a separate experiment analogous to **B** latently-infected or mock-infected cells were treated with the indicated concentrations of IL-15 for 16 hours. The percentage of HIV-Gag^+^ cells (gated as in **B**) is plotted against the concentration of IL-15 (red circles = infected, green squares = uninfected). **D.** Latently-infected cells were stimulated with the indicated LRAs for 36 hours and HIV p24 in supernatants was quantified by ELISA. Shown are background-subtracted mean ± SEM values (duplicates). P-values were calculated by ANOVA with Holm-Sidak’s multiple comparison test (comparing each condition with no treatment [No Tx]) * p < 0.05, ** p < 0.01.

**Fig 2:**
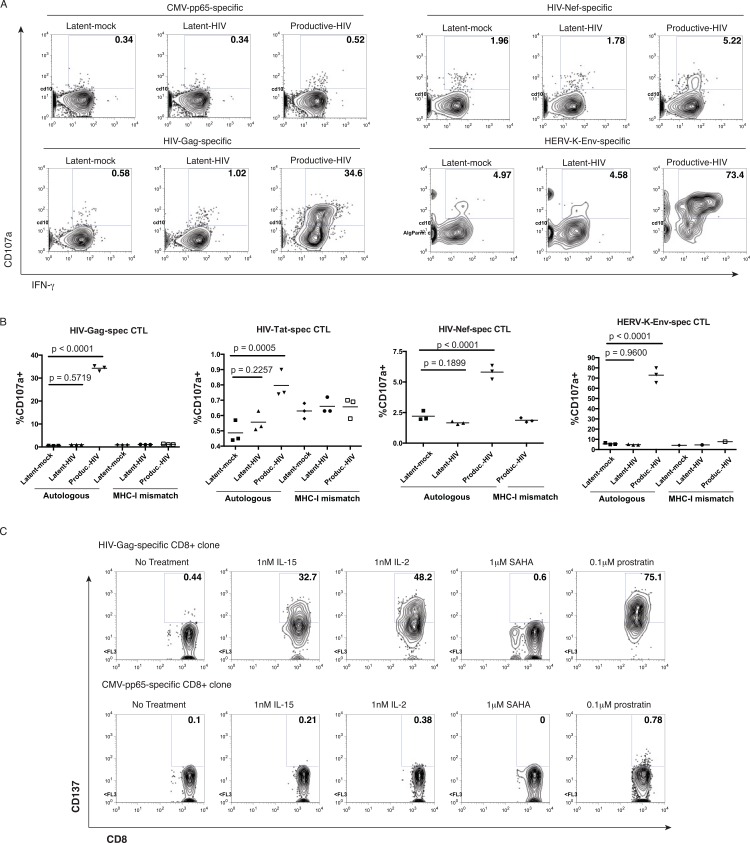
Recognition of latently-infected primary CD4^+^ T-cells by virus-specific CD8^+^ T-cell clones following exposure to candidate LRAs. HIV-, CMV- and HERV-K-specific CD8^+^ T-cell clones were derived from the HIV-infected participant OM9. Latently-infected and productively-infected target cells were prepared and characterized as in **Fig 1A**. The designated CD8^+^ T-cell clones were co-cultured with the indicated target CD4^+^ T-cells (autologous) immediately after the depletion of activated cells. Cells were stained and fixed after 16 hour co-cultures. **A.** Shown are flow cytometry data gated on CD3^+^CD8^+^ lymphocytes and depicting CD107a (degranulation)–y-axis, by IFN-γ –x-axis. Latently-infected cells did not induce CD107a exposure. **B.** Summary flow cytometry data of the same experiment depicted in **A**. P-values were calculated by ANOVA with Holm-Sidak’s multiple comparison test (comparing each condition with latent-mock). All conditions/replicates tested are shown. Latent-mock and latent-HIV conditions of MHC-I mismatch were omitted from the HIV-Nef-spec CD8^+^ T-cells due to insufficient cell numbers, as were replicates of MHC-I mismatch conditions for the HERV-K-Env-specific CD8^+^ T-cell clone. CD107a exposure by an HIV-specific CD8^+^ T-cell clone was only induced by productive HIV infection. **C**. In a separate experiment, latently-infected target cells were prepared in the same manner as **A,** rested for 72 hours, and then combined with an autologous HIV-Gag-specific CD8^+^ T-cell clone (upper panels) or an autologous CMV-pp65-specific CD8^+^ T-cell clone (lower panels) for a 24 hour co-culture period. Candidate latency-reversing drugs were added as indicated above the corresponding panels, and left in for the duration of co-cultures. Shown are flow cytometry data gated on CD3^+^CD8^+^ lymphocytes and depicting CD137 (4-1BB)–y-axis by CD8 x-axis. CD137 expression by an HIV-specific CD8^+^ T-cell clone was induced following treatment with IL-15, IL-2, and prostratin, but not SAHA.

To test this, we generated a panel of three representative HIV-specific CD8^+^ T-cell clones targeting Gag, Tat, and Nef, one CMV-pp65-specific clone, and one human endogenous retrovirus K (HERV-K)-specific CD8^+^ T-cell clone from HIV-infected subjects. We have previously demonstrated that this HERV-K-specific CD8^+^ T-cell clone specifically responds to and eliminates HIV-infected target cells due to the induction of HERV-K antigen expression by HIV[27,28]. We utilized a primary cell model of HIV latency where CCL19-treated resting CD4^+^ T-cells were directly infected with HIV followed by removal of activated cells. This is a modified version of a previously described model, commonly referred to as the “Lewin model”[16], with the distinction that the Lewin model depletes activated CD4+ T-cells prior to HIV infection, whereas we depleted activated CD4+ T-cells after HIV infection, immediately prior to use in downstream assays. These target cells showed little to no HIV-Gag expression in resting CD4^+^ T-cells **(Fig 1A),** but expressed Gag protein detectable by intracellular staining following treatment with LRAs including IL-15 and prostratin **(Fig 1B and 1C).** HIV-Gag staining in cells which were stimulated with anti-CD3/CD28 and then infected with HIV is shown for comparison **(Fig 1A).** A subset of LRAs were further capable of inducing p24 protein detectable in the supernatant of treated latently-infected cells (**Fig 1D**). These latently HIV-infected or mock-infected target cells, as well as productively HIV-infected cells (anti-CD3/CD28 stimulated), were co-cultured with autologous CD8^+^ T-cell clones to assess the presentation of HIV antigens. CD8^+^ T-cell recognition of infected target cells was assessed by degranulation (exposure of CD107a) and by production of IFN-γ following a 16 hour co-culture. We observed that each of the HIV-specific CD8^+^ T-cell clones as well as the HERV-K-specific clone responded to productively-infected cells, while responses to latently-infected cells were not detected (**Fig 2A and 2B**). As expected, a CMV-pp65-specific clone did not respond to any of these targets. These data indicate that latently infected cells in this model are refractory to CD8^+^ T-cell recognition as measured by this assay.

### A subset of putative LRAs prime latently-infected cells for CD8^+^ T-cell recognition

We next evaluated the abilities of different LRA to sensitize these latently infected cells for CD8^+^ T-cell recognition. We established co-cultures of HIV-Gag- or CMV-pp65-specific CD8^+^ T-cell clones with autologous latently-infected target cells and candidate LRAs. In order to avoid the use of brefeldin A, which would prevent MHC-I presentation of reactivated viral antigens, we utilized CD137 as a marker of CD8^+^ T-cells that had recognized targets. CD137, also known as 4-1BB is a member of the tumor necrosis receptor family that is specifically upregulated on CD8^+^ T-cells following antigen recognition[30]. ARVs were added to cultures to prevent virus propagation. We observed little to no recognition of latently-infected cells by the HIV-Gag-specific CD8^+^ T-cell clone in the absence of LRAs over a 24 hour co-culture. (**Fig 2C, upper panel**). The addition of IL-15, IL-2, or prostratin to co-cultures (left in for duration of 24 hour co-culture periods) led to CD8^+^ T-cell recognition, as measured by upregulation of CD137 on CD8^+^ T-cells, while the addition of the histone deacetylase inhibitor (HDACi) SAHA did not (**Fig 2C, upper panel**). Upregulation of CD137 was not observed in any of these conditions on the CMV-pp65-specific CD8^+^ T-cell clone (**Fig 2C, lower panel**). Thus, IL-15, IL-2, and prostratin induced the expression of HIV antigens from latently-infected cells in this primary cell model, as indicated by specific recognition by HIV-specific CD8^+^ T-cells.

**Fig 3:**
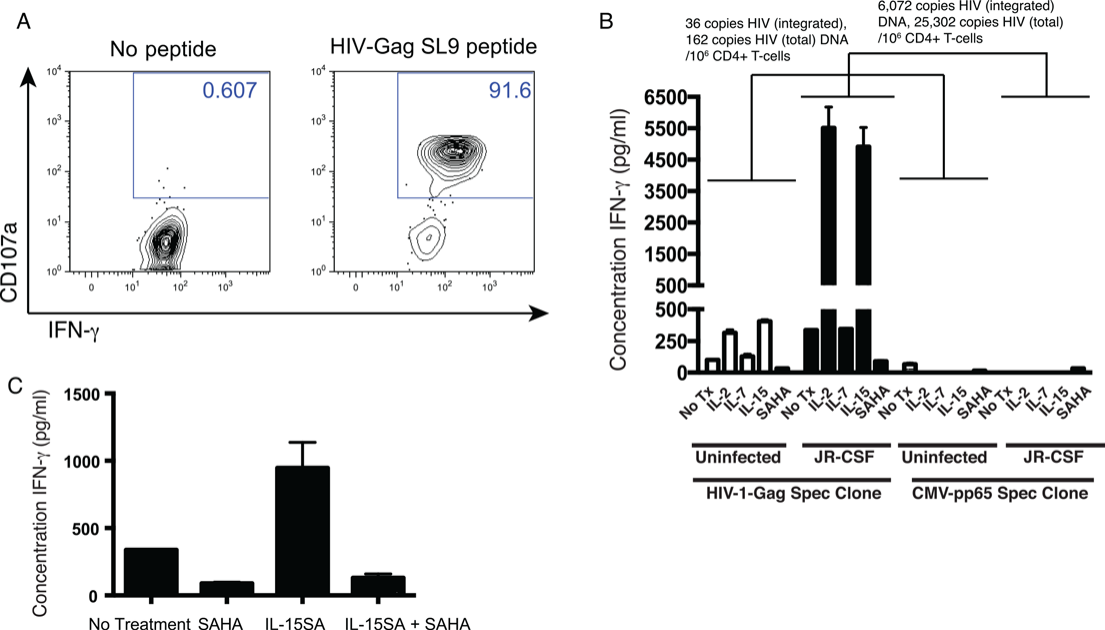
A subset of latency-reversing agents prime latently-infected CD4^+^ T-cells for CD8^+^ T-cell recognition in a continuous co-culture assay. **A.** An HIV-Gag-SLYNTVATL (SL9) specific CD8^+^ T-cell clone was isolated from subject OM9. To confirm specificity, this clone was co-cultured with an autologous B lymphoblastoid cell line (BLCL) that had been pulsed with 1 μg/ml of SL9 peptide or with an unpulsed control. Shown are flow cytometry data gated on CD8^+^ lymphocytes, depicting CD107a staining (degranulation)–y-axis by IFN-γ –x-axis. These data indicate that the CD8^+^ T-cell clone to be used in subsequent panels was highly specific. **B**. CD4^+^ T-cells latently infected with HIV-JR-CSF, or mock infected, as described in Fig 1, using leukapheresis material autologous to the CD8^+^ T-cell clone in **A.** Total and integrated HIV DNA were quantified by qPCR. These cells were treated with the indicated drugs at the following concentrations: IL-2 1.3 nM, IL-7 1 nM, IL-15 1.4 nM or SAHA 500 nM for 72 hours in the presence of nevirapine. These concentrations of IL-2, IL-7, and IL-15 equate to 20 ng/ml, and were selected based on concentrations used in previous studies of latency reversal [32,33]. Target cells were then co-cultured with the HIV-Gag-specific CD8^+^ T-cell clone from **A** for an additional 72 hours. Shown are IFN-γ levels quantified in supernatants (mean ± SEM). These results indicate that IL-2 and IL-15 primed latently-infected cells for recognition by CD8^+^ T-cells. **C.** An experiment was setup in an identical manner to **B**, co-culturing with an HIV-Gag-SL9-specific CD8^+^ T-cell clone in the presence of the indicated single of combinations of drugs at the following concentrations: SAHA 500 nM; IL-15SA 1.4 nM. Shown are mean ± IFN-γ quantifications (ELISA).

To confirm and extend these results to additional LRA candidates, we employed an assay that utilizes IFN-γ production from co-cultures as a measure of CD8^+^ T-cell recognition. Two variations on this assay were tested, one where LRAs were added for the duration of the co-cultures, and a second where targets were pre-treated with LRAs, which were washed out prior to co-culture in order to minimize potential effects of these agents on effector cells[31]. Results from an experiment without a wash-out step are presented in **Fig 3**. We selected an HIV-Gag-SLYNTVATL (SL9) specific CD8^+^ T-cell clone that exhibited robust degranulation (CD107a) and IFN-γ production in response to its cognate peptide, and used autologous CD4^+^ T-cells from the infected donor (**Fig 3A**). Levels of cell associated HIV DNA were measured in target cells prior to co-culture with effector cells, and in the depicted experiment were determined to be 36 integrated copies/10^6^ CD4^+^ T-cells and 162 total copies/10^6^ CD4^+^ T-cells in uninfected (i.e: non-super-infected cells, represents natural reservoir in this patient sample) and 6,072 integrated copies/10^6^ CD4^+^ T-cells and 25,302 total copies/10^6^ CD4^+^ T-cells in JR-CSF-infected cells. We observed a small amount of IFN-γ production by HIV-Gag-specific T-cell clones that were co-cultured with HIV-infected target cells in the absence of LRAs, suggesting that some recognition of latently-infected cells is detectable using this more sensitive readout (relative to CD137 upregulation) in this primary cell model (**Fig 3B**, “JR-CSF/No Tx”). This baseline level of IFN-γ production was substantially enhanced in co-cultures that were treated with IL-2 or IL-15, but not in those treated with IL-7 or SAHA (**Fig 3B**). A lack of IFN-γ production was observed from the CMV-pp65-specific T-cell clone in all co-culture conditions, further supporting that the effects observed with the HIV-Gag-specific T-cell clone were driven by HIV antigen recognition. Interestingly, we observed a similar pattern of IFN-γ production by the HIV-Gag-specific T-cell clone in response to uninfected (i.e: non-super-infected) cells as compared to JR-CSF-infected cells, though much lesser in magnitude, with IL-2 and IL-15 treatment associated with elevated levels of IFN-γ (p < 0.001 for comparisons between both IL-2 and IL-15 with no treatment. This could be interpreted either as indicating that these LRAs induce some level of spontaneous release of IFN-γ or, since target cells were from an ARV-treated HIV-infected subject, as indicating low-level recognition of the natural HIV reservoir following LRA treatment. Some initial support for the latter hypothesis can be drawn from the observation that in this same experiment, treatment with IL-2 and IL-15 was not associated with spontaneous cytokine production from the CMV-pp65-specific T-cell clone. More in depth analysis supporting this latter hypothesis are presented below.

In this experiment, we also observed a lesser amount of IFN-γ production in co-cultures of the HIV-Gag-specific T-cell clone and infected targets in the presence of SAHA as compared to the no treatment control. This is consistent with our previous observations that SAHA suppresses IFN-γ production from T-cells in experiments where the drug is left in[31]. We then tested the combination of an IL-15 superagonist (comprising IL-15 bound to a soluble form of the IL-15 receptor α chain) with SAHA. In line with the previous experiment, we observed that SAHA treatment resulted in diminished IFN-γ production as compared to no treatment, while the IL-15SA drove enhanced IFN-γ production. The latter effect was abrogated when IL-15SA was combined with SAHA, supporting that this drug suppressed IFN-γ production from the T-cell clone (**Fig 3C**). Thus, the results of these experiments represent the net effects of candidate LRAs on the target cells (latency reversal) and on the T-cell clone (modulation of function).

**Fig 4:**
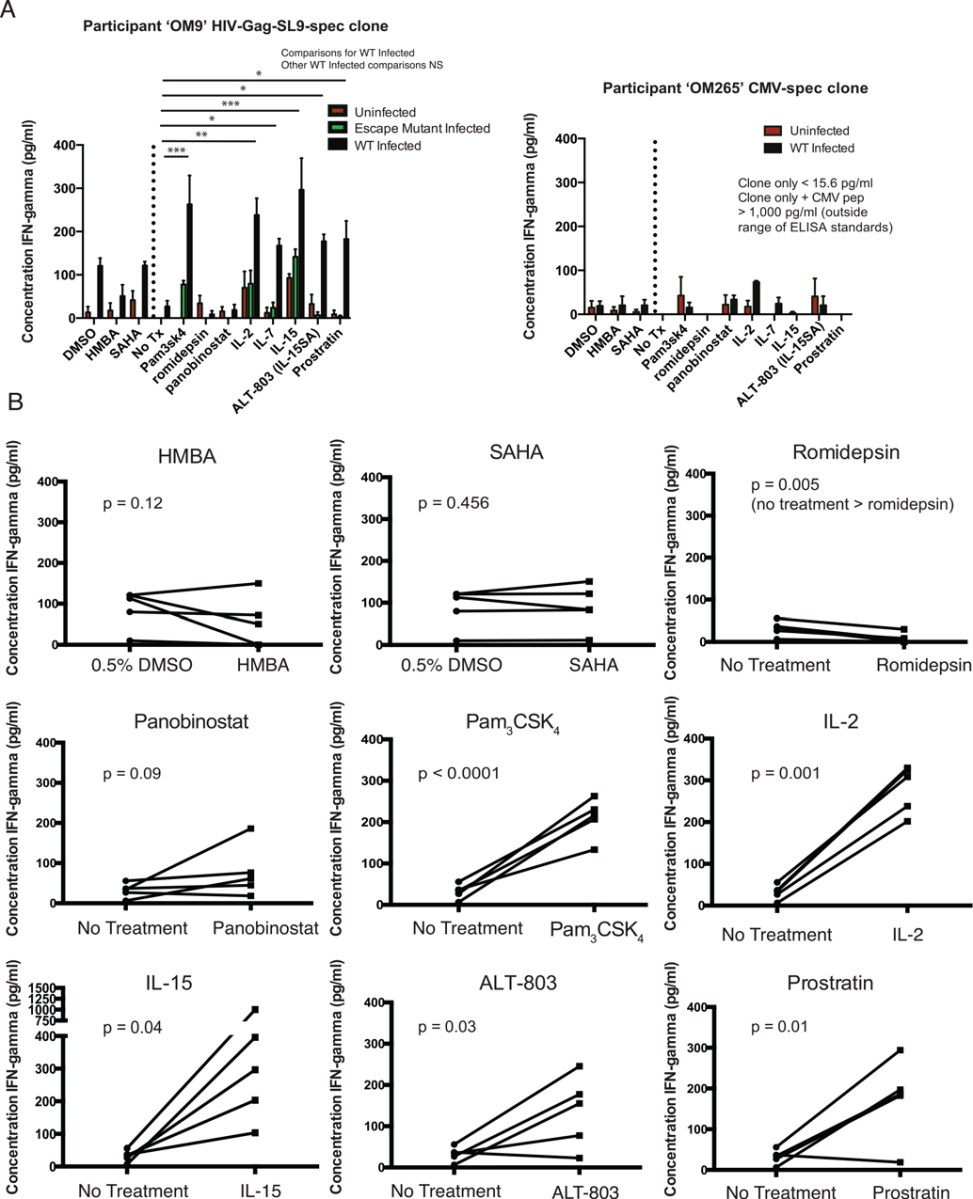
A subset of latency-reversing agents primes latently-infected CD4^+^ T-cells for CD8^+^ T-cell recognition in a pulse-wash assay. Latently-infected resting CD4^+^ T-cell targets were prepared as in Fig 1, using cells from three different HIV-infected subjects: OM9, OM292, OM265. Cells were infected with HIV-LAI, HIV-LAI-SL9-escape (three mutations in epitope), or mock infected. Specificities of the following CD8^+^ T-cell clones were confirmed by degranulation assays (CD107a): HIV-Gag-RV9 (OM292), HIV-Env-VL9 (OM292), HIV-Gag-SL9 (OM265), HIV-Pol-TY9 (OM265), CMV pool (OM265), and HIV-Gag-SL9 (OM9). Target cells were plated in ARVs and treated with the following concentrations of putative LRA for 56 hours: no treatment, 0.5% DMSO, 0.5 mM HMBA in 0.5% DMSO, 6.7 μM Pam_3_CSK_4_, 500 nM SAHA in 0.5% DMSO, 25 nM romidepsin, 25 nM panobinostat, 1.3 nM IL-2, 1 nM IL-7, 1.4 nM IL-15, 1.4 nM **ALT-803**, 39 nM prostratin. These concentrations of IL-2, IL-7, and IL-15 equate to 20 ng/ml, and were selected based on concentrations used in previous studies of latency reversal [32,33]. The concentration of ALT-803 was set equimolar to IL-15. Concentrations of the HDAC inhibitors were selected based on previous reports indicating latency reversal without substantial toxicity to resting CD4^+^ T-cells [17,31,37]. The concentration of prostratin was selected to be on the low end of its active range against HIV latency to challenge the sensitivity of the assay. Target cells were then washed 3x to remove LRAs, and CD8^+^ T-cell clones were added. Following a 16 hour CD8^+^ T-cell + target cell co-culture, supernatants were harvested for IFN-γ ELISA. **A.** Shown are mean ± SEM IFN-γ ELISA data for an HIV-Gag-specific CD8^+^ T-cell clone (left) and a CMV-pp65-specific CD8^+^ T-cell clones (right). In each panel, the drugs to the left of the dotted vertical line were dissolved in 0.5% DMSO and thus are comparable to the DMSO control while those to the right of the dotted vertical line are in PBS (Il-2, IL-7, IL-15, ALT-803, Pam_3_CSK_4_) or < 0.01% DMSO (romidepsin, panobinostat, prostratin) and thus are comparable to the no treatment (No Tx) control. P values refer to differences in the wild-type infected condition and were calculated by ANOVA with Holm-Sidak’s multiple comparison test. The results indicate that a subset of LRAs prime latently-infected cells for recognition by this representative CD8^+^ T-cell clone. **B.** Shown are summary ELISA data with each line representing a single CD8^+^ T-cell clone tested against autologous target cells with the indicated LRAs or controls. P values were calculated by paired t-tests. The results indicate that a subset of LRAs consistently prime latently-infected cells for recognition by a panel of CD8^+^ T-cell clones.

To isolate the effects of candidate LRAs on latently-infected cells, we modified our experimental protocol to include a step to wash out drugs prior to the addition of CD8^+^ T-cells. Resting CD4^+^ T-cells were cultured with LRAs for 72 hours, and were then washed three times to remove the drugs. Washed cells were then co-cultured with HIV-specific or CMV-specific CD8^+^ T-cell clones for 18 hours, and IFN-γ in the supernatants was measured by ELISA. We added additional putative LRAs to this experiment, including: Pam_3_CSK_4_, a TLR-2 agonist previously shown to reverse HIV latency in a post-activation primary cell model[34]; the HDAC inhibitors panobinostat and romidepsin;, and hexamethylbisacetamide (HMBA) which has been reported to induce the expression of latent HIV through chromatin remodeling[35,36]. In this and subsequent experiments we also utilize the drug ALT-803, an IL-15 superagonist that is currently in clinical trials (see discussion). This change from using IL-15SA to ALT-803 was motivated by a desire to ensure that any results could be rapidly translated to the clinic. Using an HIV-Gag SLYNTVATL-specific CD8^+^ T-cell clone, we observed that IL-2, IL-7, IL-15, ALT-803, Pam_3_CSK_4,_ and prostratin each facilitated CD8^+^ T-cell recognition of cells that had been latently-infected by the HIV molecular clone JR-CSF, which contains the targeted epitope, while panobinostat, HMBA, SAHA, and romidepsin were not associated with CD8^+^ T-cell recognition (**Fig 4A**). The specificity of the assay was supported by the lack of appreciable recognition of target cells by a CMV-specific CD8^+^ T-cell clone following treatment with any of the LRAs tested **(Fig 4A, right panel).** As in the previous experiment in which drugs were not washed out (**Fig 3**), we did observe some recognition of cells not infected with JR-CSF, and also of cells infected with an escape mutation in the targeted SLYNTVATL epitope, following treatment with a subset of LRAs, including IL-2, and IL-15. Again, this was not observed with the CMV-specific T-cell clone, suggesting recognition of the autologous patient reservoir following treatment with these LRAs rather than non-specific production of IFN-γ in response to these cytokines. This is further tested below.

Applying our latency reversal T-cell recognition assay to a set of 5 HIV-specific CD8+ T cell clones (with autologous targets) we observed that IL-2, IL-15, ALT-803, Pam_3_CSK_4_, and prostratin primed infected targets for CD8^+^ T-cell recognition, while HMBA, SAHA, and panobinostat did not **(Fig 4B)**. We observed significantly less IFN-γ production from CD8^+^ T-cell co-cultured with romidepsin-treated cells than untreated controls. We interpret this as indicating that romidepsin was released from target cells (despite washing), suppressing CD8^+^ T-cell clone function as we have previously described[31], which confounds results for this particular LRA. Together, these data identify IL-2, IL-15, ALT-803, Pam_3_CSK_4_, and prostratin as LRAs that consistently prime latently-infected cells for HIV-specific CD8^+^ T-cell recognition in this *in vitro* model system.

### Detection of ALT-803 reactivated cells from the natural reservoir by HIV-specific CD8+ T-cells

In the experiments depicted in **Figs 3 and 4** we observed low levels of HIV-specific T-cell recognition of autologous CD4^+^ T-cells that had not been superinfected with HIV, following treatment with a subset of latency-reversal agents. We hypothesized that this represented recognition of the natural patient HIV reservoir rather than non-specific LRA-induced IFN-γ production, and this was supported by the observation that a parallel effect was not observed with CMV-specific T-cell clones. To further test this hypothesis, we next determined the ability of an HLA-A02-restricted HIV-Gag-SLYNTVATL-specific CD8^+^ T-cell clone to sense the latency reversal in the natural reservoir in *ex vivo* CD4^+^ T-cells from HLA-A02^+^ and HLA-A02^-^ donors, following treatment with an LRA. As these experiments are very intensive in terms of cell numbers, we focused on a single LRA. We opted to focus on ALT-803 having shown that this functions as an LRA, and because this drug is a viable clinical candidate with an established safety record in cancer patients, and a clinical trial is planned for ARV-treated HIV-infected patients (NCT02191098).

**Fig 5:**
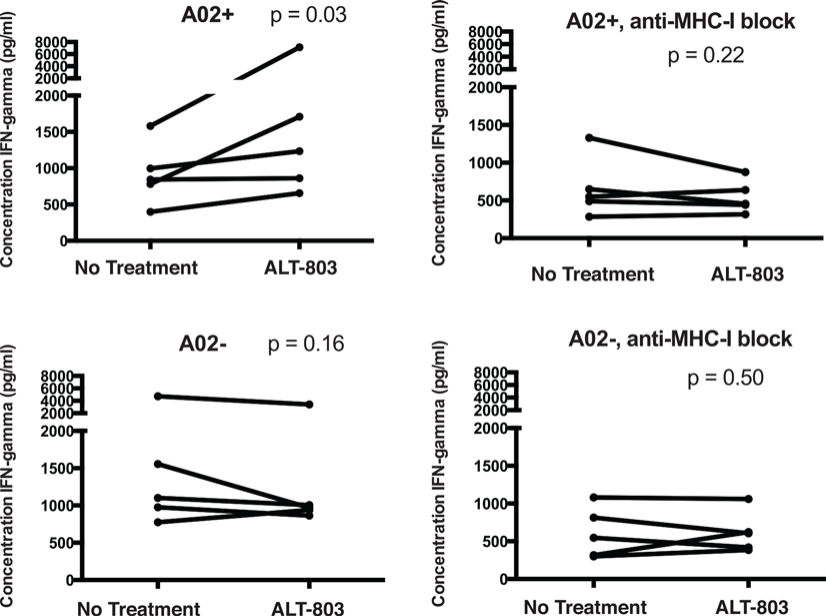
HIV-specific CD8^+^ T-cells sense ALT-803 mediated viral reactivation from the natural HIV reservoir. PBMC from 5 HLA-A02^+^ and 5 HLA-A02^-^ ARV-treated HIV-infected subjects were depleted of CD8^+^ T-cells, and of cells expressing CD69, CD25, or HLA-DR and then stimulated with 1.4 nM ALT-803 or maintained as untreated controls for 72 hours in the presence of ARVs. These target cells were then washed and co-cultured with an HIV-Gag-SL9-specific CD8^+^ T-cell clone (HLA-A02 restricted) for 18 hours with the addition of either an anti-MHC-I blocking antibody or an isotype control. Shown are ELISA data measuring concentrations of IFN-γ in supernatants at the end of this co-culture period. Each condition was tested in duplicate and mean values were plotted. P values were calculated by one-tailed Wilcoxon matched pairs tests. The results indicate that ALT-803 primes latently-infected cells from the natural reservoir for recognition by an HIV-specific CD8^+^ T-cell clone.

Target cells containing natural latently-infected cells, were prepared from HIV^+^ patient PBMCs by depleting activated cells (expressing CD69 or HLA-DR) and CD8^+^ T-cells, then treating with or without 0.7 nM ALT-803. The CD8^+^ T-cell clone was then cultured with these targets in the presence or absence of an anti-MHC-I blocking antibody or an isotype control. We observed significant induction of IFN-γ production from CD8^+^ T-cells co-cultured with ALT-803-treated HLA-A02+ target cells (**Fig 5**, p = 0.03). Parallel inductions were not observed in the presence of the anti-MHC-I antibody, nor following co-culture with HLA-A02- target cells. These results demonstrate the feasibility of utilizing HIV-specific CD8^+^ T-cell clones as sensors for latently reversal in the natural patient reservoir. They confirm that ALT-803 induces detectable antigen expression from these patient samples to a level that allows these cells to be targeted by HIV-specific CD8^+^ T-cells.

**Fig 6:**
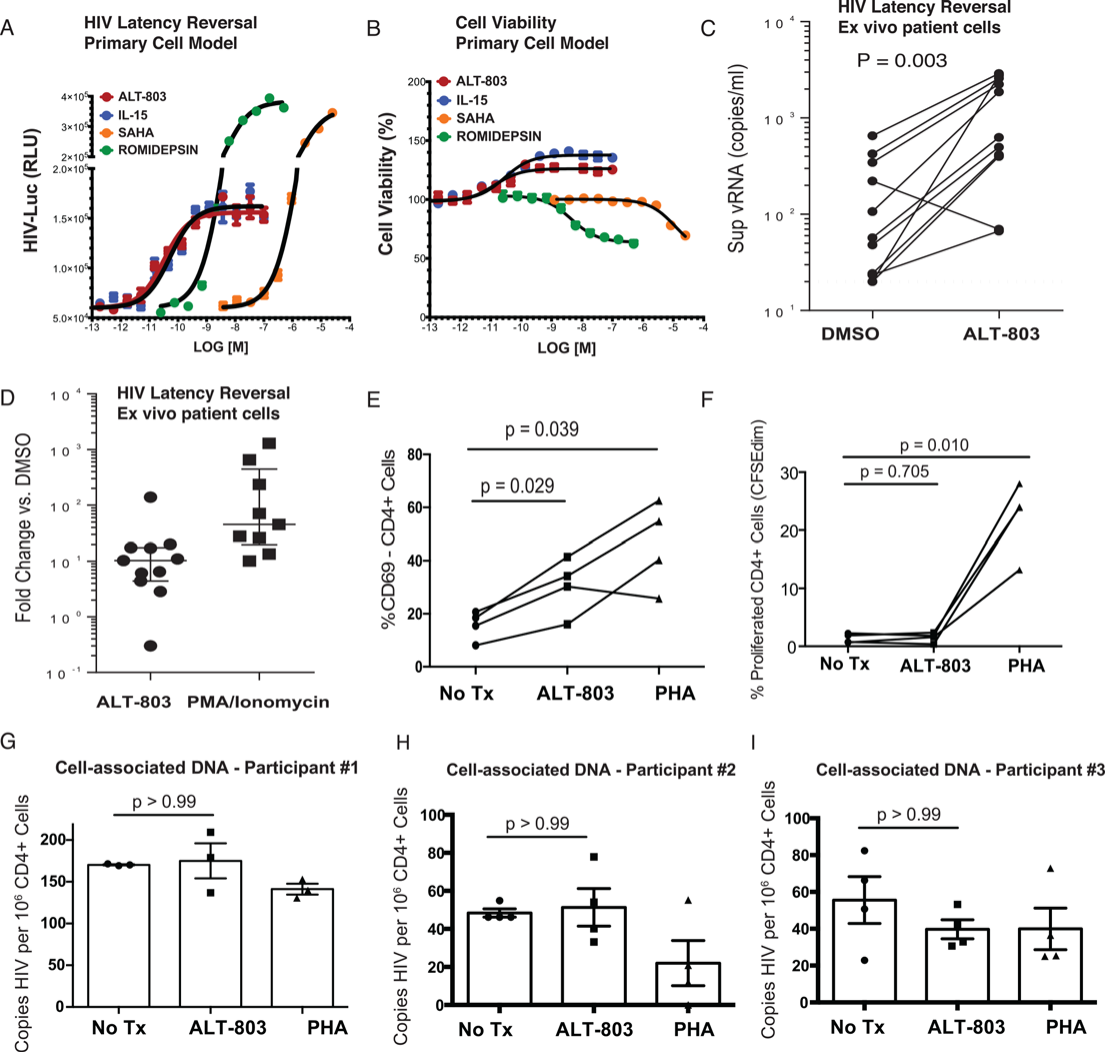
ALT-803 induces HIV transcription from both a primary cell model of latency and from *ex vivo* patient samples. Primary CD4^+^ T-cells from healthy donor PBMC were activated, infected with a luciferase reporter HIV virus, and allowed to return to a resting state. This is similar to a post-activation latency model that has been previously described [17] with additional minor modifications (see Methods). After 1 week, infected cells were treated with the agents at concentrations indicated for 2 days. **A.** Shown are luminescence values from luciferase, indicating dose-dependent HIV reactivation by each of the compounds tested. **B.** Compound associated cytotoxicity was determined in latently-infected cells in parallel with the virus activation assay using Cell Titer Glo (CTG) Dose dependent cytotoxicity was observed with SAHA and romidepsin but not with IL-15 or ALT-803. **C and D.** PBMC were isolated from HIV-infected subjects who had been suppressed by ART for at least 2 years. Cultures were maintained in the presence of ARVs. Either 1 nM of ALT-803 or 5 ng/ml PMA + 500 ng/ml ionomycin were added for 7 days. HIV activation was measured by quantitating viral RNA in cell-free supernatant using COBAS qPCR. **C.** The geometric mean of viral copies/ml for each donor are indicated for control (DMSO) and ALT-803 treated wells, following 7 days of treatment. **D**. The fold HIV activations of all donors are plotted as the ratio of the viral copies/ml for ALT-803 treated or PMA/ionomycin treated to control wells. These results indicate that ALT-803 reactivates virus from the natural patient reservoir, though to a lesser degree than PMA/ionomycin. **E-I.** Cryopreserved CD4^+^ T-cells from ARV-treated HIV-infected subjects were thawed, CFSE-labeled, and treated with either 1 nM ALT-803 or 200 ng/ml of PHA for 7 days in the presence of ARVs. **E**. Activation of CD4^+^ T-cells within PBMCs was measured at day 7 by flow cytometry, gating on CD3^+^CD4^+^ T-cells and assessing CD69 expression. Each line represents a different subject. P values were calculated by RM one-way ANOVA with Holm-Sidak’s multiple comparison test. **F**. Proliferation of CD4+ T-cells within PBCs was measured at day 7 by flow, gating on CD3^+^CD4^+^ T-cells and assessing proliferation as %CFSE^dim^ cells. P values were calculated by RM one-way ANOVA with Holm-Sidak’s multiple comparison test. G-I. Cell associated DNA was isolated from purified CD4^+^ T-cells following 6 days of stimulation. Absolute copies of HIV-Gag and RPP30 were determined by droplet digital PCR and used to calculate copies of HIV per 10^6^ CD4^+^ T-cells. Shown are results from three different subjects. Quantifications were determined in triplicate (**G**) or quadruplicate (**H & I**). Means and SEM are shown and the P value was calculated by one-way ANOVA with Holm-Sidak’s multiple comparison test.

We next confirmed HIV latency-reversal by ALT-803 using more conventional assays. We first utilized a primary cell model of viral latency, where naïve CD4^+^ T-cells were activated with anti-CD3/CD28 magnetic beads, infected with a single round recombinant *env* ^-^ HIV virus containing a firefly luciferase reporter gene (*fluc*) in place of *nef*, and allowed to return to a quiescent state[17]. These resting cells were then treated with LRAs, and viral reactivation was measured by luciferase activity. We observed that both ALT-803 and IL-15 reversed HIV latency in this model, with maximal effects observed with as little as 1 nM of each agent (**Fig 6A**). Latency reversal was also observed with higher concentrations of SAHA or romidepsin as has been previously reported[17]. The cytotoxicity of these compounds in these cells was tested in parallel using a luminescent cell viability assay (see Methods). Whereas the concentrations of romidepsin and SAHA required for latency reversal were associated with cytotoxicity, we did not observe any loss in cell viability with either IL-15 or ALT-803 (Fig 6B).

We next tested the ability of ALT-803 to reactive latent virus from *ex vivo* peripheral blood mononuclear cells (PBMCs) from ARV-treated HIV-infected subjects. In PBMCs from 10 out of the 11 ARV-treated HIV-infected participants that were tested, ALT-803 at 1 nM induced significantly higher levels of supernatant viral RNA compared to the DMSO control after 7 days of culture (Fig 6C). Overall, ALT-803 induced HIV at a median of 10.4-fold compared to DMSO (Fig 6D), a level that was 14.4% of stimulation with the mitogen PMA plus ionomycin (Fig 6D). These data corroborate our CD8^+^ T-cell-based assays in identifying ALT-803 as an LRA. The treatment conditions associated with latency reversal resulted in a modest amount of activation of CD4^+^ T-cells as measured by CD69 upregulation (**Fig 6E**), but did not result in detectable proliferation of CD4^+^ T-cells (**Fig 6F**), nor in expansion of the viral reservoir as measured by cell-associated HIV DNA (**Fig 6G–6I**). Thus, *in vitro*, ALT-803 reactivates HIV expression from natural patient-derived reservoirs at conditions associated with only modest amounts of T-cell activation and no induction of proliferation or reservoir expansion.

### ALT-803 enhances CD8^+^ T-cell killing of productively HIV-infected cells

Having isolated the effects of LRAs on priming target cells for recognition, we next sought to further prioritize candidate LRAs based their impact on CD8^+^ T-cell function. In previous work, we demonstrated that the HDAC inhibitors panobinostat and romidepsin can impair multiple functions of HIV-specific CD8^+^ T-cells *in vitro*, including IFN-γ production and killing ability [31]. In that same study, we had observed that IL-15 and ALT-803 enhanced HIV-specific T-cell responses by *ex vivo* IFN-γ ELISPOT. Thus, it is likely that the high levels of IFN-γ production observed in **Fig 3,** when IL-15 was added to co-cultures of latently-infected cells and HIV-specific CD8^+^ T-cell clones, was the net result of both the induction of antigen expression and the enhancement CD8^+^ T-cell function.

**Fig 7:**
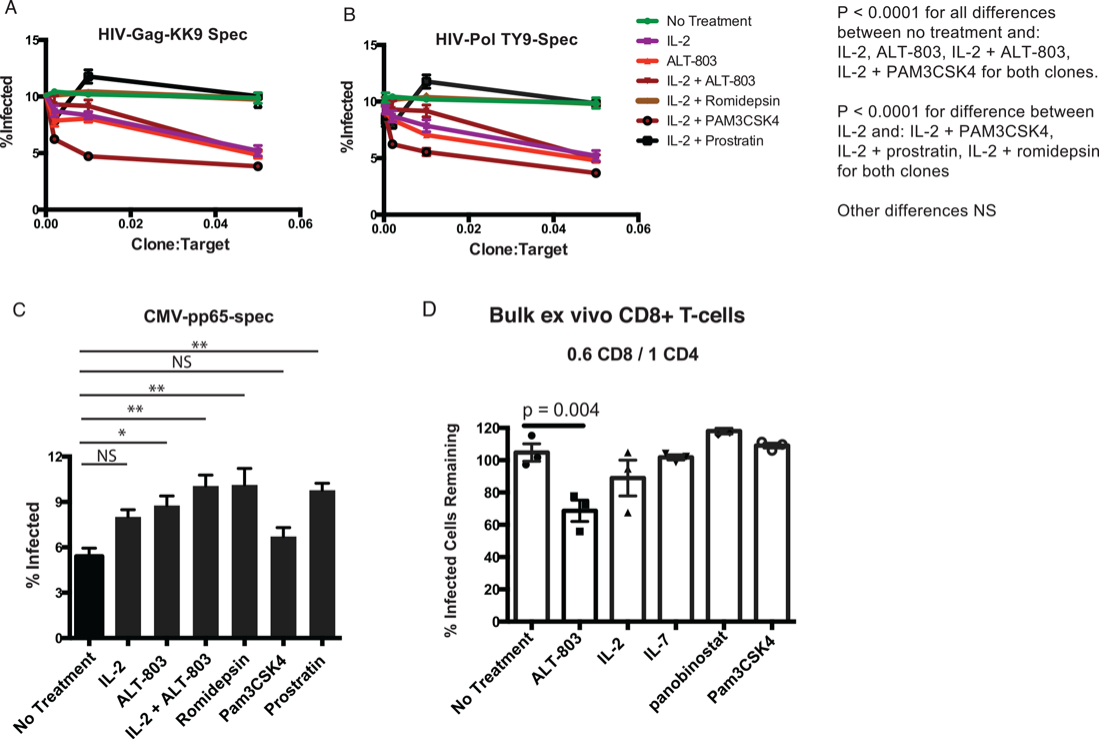
Effects of latency-reversing agents on CD8^+^ T-cell elimination of HIV-infected target cells. **A—C.** CD4^+^ T-cells were enriched from HIV-infected subjects, stimulated with anti-CD3/anti-CD28 for 48 hours, and infected with HIV-LAI. Three different CD8^+^ T-cell clones (HIV-Gag-KK9-specific, HIV-Pol-TY9-specific, and CMV-pp65-specific) were washed and then cultured in the presence of: i) RPMI-10 ii) RPMI-10 + 50 U/ml IL-2 iii) RPMI-10 + 1.4 nM ALT-803 iv) RPMI-10 + 50 U/ml IL-2 + 1.4 nM ALT-803 v) RPMI-10 + 50 U/ml IL-2 + 6.6 μM Pam_3_CSK_4_ vi) RPMI-10 + 50 U/ml IL-2 + 25 nM romidepsin vii) RPMI-10 + 50 U/ml IL-2 + 390 nM prostratin. Cells were cultured 60 hours. For the romidepsin treatment, cells were washed after 3 hours and medium was replaced with RPMI-10 + 50U/ml IL-2 for the remaining 57 hours. For the prostratin treatment cells were washed after 40 hours and given a 20 hour ‘rest’ in RPMI-10 + 50U/ml IL-2. For all other conditions LRAs were left in throughout the 60 hours and then washed repeatedly. CD8^+^ T-cell clones were then co-cultured with autologous HIV-infected target cells at the indicated clone:target ratios for 16 hours. Levels of infection were assessed by flow cytometry by gating on CD4^+^CD8^-^ viable lymphocytes and then measuring %HIV-Gag^+^CD4dim (%Infected, y-axis). All conditions were tested in triplicate. Shown are means ± SEM. P values were calculated by 2-way ANOVA with Tukey’s multiple comparison test, comparing each condition to every other condition. For both clones, P < 0.0001 for all comparisons except for the following non-significant cases: i) no treatment vs IL-2 + ALT-803 ii) no treatment vs IL-2 + prostratin iii) IL-2 vs ALT-803 iv) IL-2 vs IL-2 + ALT-803 v) ALT-803 vs IL-2 + ALT-803 vi) IL-2 + romidpesin vs IL-2 + prostratin. The results showed that IL-2, ALT-803, and Pam_3_CSK_4_ enhance killing of infected cells by CD8^+^ T-cell clones, whereas prostratin impaired killing. Non-specific killing was not observed with the CMV-pp65 **D**. CD4^+^ T-cells from an ARV-treated HIV-infected subject were stimulated with anti-CD3/anti-CD28 for 48 hours, and infected with HIV-LAI. Autologous bulk CD8^+^ T-cells were enriched by negative selection and cultured with the indicated LRAs for 16 hours at the following concentrations: ALT-803–1.4 nM, IL-2–1.3 nM, IL-7–1 nM, panobinostat– 20 nM, Pam_3_CSK_4_−3.3 μM. For ALT-803, IL-2, IL-7, and Pam_3_CSK_4_ LRAs were left in throughout the co-culture period. For panobinostat, cells were treated with LRAs for 3 hours and then drugs were washed out for remaining co-culture period. In either case, bulk CD8^+^ T-cells were washed 3 additional times prior to co-culture with autologous infected CD4^+^ T-cells for 6 hours. Cells were then stained with viability dye and antibodies to CD4, CD3, CD8, and HIV-Gag (intracellularly) and analyzed by flow cytometry. Shown are mean ± SEM of % infected cells remaining (normalized to no CD8^+^ T-cell control). Statistical significance was calculated by ANOVA with Dunnett’s multiple comparisons test. These data shown that ALT-803 enhanced the abilities of *ex vivo* CD8+ T-cells to kill HIV-infected cells.

To clarify this possibility, we directly assessed the impact of LRAs on the abilities of CD8^+^ T-cell clones to kill productively infected cells. The T-cell clones were maintained in IL-2, thus most LRAs were tested in combination with this cytokine. Following 16 hours of culture in the absence of IL-2, CD8^+^ T-cells exhibited negligible abilities to kill infected targets (**Fig 7A and 7B**). Both IL-2 and ALT-803 were able to enhance CD8^+^ T-cell function resulting in dose-dependent elimination of infected targets, though no additive effect with both compounds was observed. The additions of either prostratin or romidepsin to IL-2 abrogated CD8+ T-cell killing, while Pam_3_CSK_4_ enhanced killing (**Fig 7A and 7B**).

An important caveat of the above experiment is that CD8^+^ T-cell clones are grown in IL-2, and thus the ‘enhanced’ elimination observed with IL-2 and ALT-803 may be due more to the effects of cytokine deprivation in the no treatment condition. To move towards a more physiologically relevant scenario, we tested the impact of LRAs on killing of infected cells by bulk autologous *ex vivo* CD8^+^ T-cells in the absence of IL-2. CD4^+^ T-cells from an HIV-infected subject who initiated therapy in acute infection were enriched, activated, and infected with HIV-LAI. Autologous CD8^+^ T-cells were treated with LRAs at the indicated concentrations for 16 hours. For the HDAC inhibitor panobinostat–which we have previously associated with impairment of T-cell function [31]–we washed drug out after 3 hours to give cells 13 hours to ‘recover’ prior to co-culture with target cells. The other LRAs were left in for the full 16 hours. After washing out LRAs, CD8^+^ T-cells were co-cultured with CD4^+^ T-cells at an effector:target ratio of 0.6 CD8^+^ T-cells:1 CD4^+^ T-cell. The percentages of viable HIV-infected cells in the target CD4^+^ T-cell populations were then measured by flow cytometry. We observed a lack of significant killing of infected target cells by CD8^+^ T-cells that had not been co-cultured with LRAs (**Fig 7C**). In contrast, we observed significant elimination of infected cells by bulk CD8^+^ T-cells that had been cultured with ALT-803 (mean ± SEM; no treatment 108.5 ± 5.4%, ALT-803 75.0 ± 6.6%, P = 0.004). No significant enhancement of killing was observed with the other LRAs tested. These results highlight ALT-803 as an LRA with the ability to enhance elimination of HIV-infected cells by primary CD8^+^ T-cells, when added at a concentration of 1.7 nM. This concentration is likely to be pharmacologically achievable, as non-human primates were found to tolerate ALT-803 at a C_max_ of 25 nM (Rhode *et al*. in press). Whereas Pam_3_CSK_4_ strongly enhanced the elimination of HIV-infected cells by HIV-specific CD8^+^ T-cell clones, no effect was observed with *ex vivo* CD8^+^ T-cells (see discussion).

## Discussion

Our study is based on the premise that, in facing the challenge of eradicating HIV infection, effectively harnessing the host immune response is likely to be paramount. From this vantage, immune effectors should be integrated into early stages of evaluating potential latency reversal strategies, with the optimal LRA being one that achieves a sufficient threshold of reactivation (submaximal) to expose infected cells to the immune system, and enhances the abilities of immune effectors to eliminate these cells. In a primary cell model, we identified four compounds that exposed latently-infected cells for recognition by HIV-specific CD8^+^ T-cells. In contrast, we were not able to observe recognition of latently-infected cells following treatment with HMBA or with HDAC inhibitors. As with all negative results, the latter observation comes with caveats, such as assay sensitivity. We also acknowledge the limitations of performing these experiments in a primary CD4+ T-cell model of latency, rather than against the natural reservoir. These results should, however, contribute to the ongoing discussion regarding the degree of latency reversal that is achieved by HDAC inhibitors. Based on our data, inefficient induction of antigen presentation by HDAC inhibitors should be considered as a potential contributing factor underlying the observation that, thus far, clinical trials of these drugs have not achieved reductions in viral reservoirs.

Intriguingly, our data support that IL-15/ALT-803 and IL-2 also prime the natural reservoir in *ex vivo* CD4^+^ T-cells for recognition by autologous HIV-specific CD8^+^ T-cells. This was an unexpected result, given that, although on the order of 1,000/10^6^ resting CD4^+^ T-cells harbor an HIV provirus in a typical ARV-treated subject, only in the range of 1/10^6^ of these harbor an intact inducible virus that can re-seed infection in viral outgrowth assays[6]. If it were only this latter population that served as a source of potential antigens, it would not be plausible that these could give rise to the observed signals. A potential explanation comes from a recent study that demonstrated the presence of ‘intact non-induced’ HIV proviruses that do not re-seed infection in a single round of a viral outgrowth assay and which substantially outnumber ‘intact induced’ proviruses in the majority of subjects [38]. Additionally, it has been reported that defective proviruses containing deletions (which comprise the majority of proviruses) can be expressed as proteins[39]. Thus, we propose that the observed recognition of the natural reservoir by HIV-specific CD8^+^ T-cell clones is a result of LRA-induced antigen expression from a spectrum of provirus-harboring cells, not just those with intact inducible provirus.

Of the five agents that primed resting CD4^+^ T-cells for CD8^+^ T-cell recognition, prostratin impaired the ability of an HIV-specific T-cell clone to eliminate HIV-infected cells, while enhanced killing was observed with IL-2, ALT-803, and Pam_3_CSK_4_, highlighting the potential of each of these as LRAs in shock-and-kill HIV eradication strategies. In line with our results, each of these three agents has been shown to enhance T-cell function in multiple systems, including *in vivo* infectious disease and cancer models[40–53]. When we utilized bulk *ex vivo* CD8^+^ T-cells (rather than clones) as effectors, ALT-803 was the sole agent that significantly enhanced elimination of infected cells, and thus stands out in our study as holding particular promise for CD8^+^ T-cell based shock-and-kill HIV eradication strategies. To achieve viral reservoir reductions in patients it may be important to perform trials in subjects with potent HIV-specific CD8^+^ T-cell responses against non-escaped epitopes, for example, in subjects treated early post-infection and for a limited duration[54], or in combination with therapeutic vaccination. Future work will seek to resolve the apparent disconnect between the ability of Pam_3_CSK_4_ to potently enhance elimination of infected cells by HIV-specific CD8^+^ T-cell clones, but not by *ex vivo* CD8^+^ T-cells from the subject that we tested. We propose that this effect may have been due to the higher baseline activation state of the clones, as the TLR-2 receptor is known to be upregulated on activated CD8^+^ T-cells[55]. Pam_3_CSK_4_ may have greater ability to boost the function of *ex vivo* CD8^+^ T-cells from subjects who have been on ART for shorter durations (less resting HIV-specific CD8^+^ T-cells), or in combination with therapeutic vaccinations. Our observations, combined with previous reports indicating that Pam_3_CSK_4_ reverses CD8^+^ T-cell exhaustion and enhances both tumor and pathogen-specific T-cell responses *in vivo* supports the prioritization of this pathway for further study for CD8^+^ T-cell based shock and kill eradication strategies [40–42].

Although not a primary conclusion of the current study, the data presented in **Fig 7** does touch on the potential for the HDAC inhibitor romidepsin to impair T-cell function *in vitro*, as reported by us previously[31]. In our previous study, the HDAC inhibitor vorinostat exhibited little to no inhibition of T-cell function *in vitro* (depending on the assay tested), while the effects of romidepsin were consistent and pronounced. A series of clinical studies have since reported convincing data indicating a lack of impairment of *ex* vivo HIV-specific T-cell responses following *in vivo* administration of vorinostat to ARV-treated subjects[21,56]. Further work is required to determine whether the romidepsin impairs HIV-specific T-cell responses *in vivo*. In a recent study, trends towards decreases in frequencies of HIV-specific IFN-γ producing cells were observed in romidepsin-treated individuals, however these were not statistically significant with n = 5. As with our previous work, our observations are limited to *in vitro* assays and we hope will serve to motivate further CD8^+^ T-cell measures in ongoing *in vivo* studies.

ALT-803 is an IL-15 superagonist complex, comprised of a human IL-15N72D mutein bound to the human IL-15RαSu/Fc[57]. Complexes of IL-15 bound to the IL-15Rα (IL-15:IL-15Rα) exhibit increased affinity of the IL-15 to the IL-2Rβ chain expressed by natural killer (NK) and T-cells[58]. Compared with native IL-15, the soluble IL-15N72D:IL-15RαSu/Fc complex (ALT-803) has a significantly longer *in vivo* half-life (25 h vs. <40 min) and increased biological activity (>25-fold more active than IL-15)[58]. Previous *in vivo* studies have shown ALT-803 to be a potent immunostimulatory complex, promoting the activation and proliferation of NK cells and CD8^+^ T cells against cancer and infectious disease, with minimal induction of CD4^+^ T cell proliferation [46,50,52,59,60]. Currently, ALT-803 is in several clinical trials against solid and hematological tumors. (NCT01946789, NCT01885897, NCT02099539). In non-clinical studies, serum concentrations of ALT-803 up to 2.8 μg/ml (25 nM) were achieved in cynomolgus monkeys without inducing overt clinical toxicity. Evaluations are currently underway to determine whether ALT-803 can be tolerated by patients at a similar dose.

In addition to identifying specific promising compounds, our study presents a novel method that enables the screening of LRAs for CD8^+^ T-cell based shock-and-kill eradication strategies. This assay requires the identification of CD8^+^ T-cells targeting epitopes that are not escaped in the autologous reservoir, and the generation of corresponding CD8^+^ T-cell clones. Once generated, these clones can be expanded and used extensively for multiple experiments. A recent report found that agents which exhibited a lack of detectable latency-reversing activity when tested on their own against patient samples, including SAHA and panobinostat, effectively synergized with other agents when tested in combination[19]. In future work it will be important to test the abilities of such combinations to enhance priming latently-infected cells for CD8^+^ T-cell recognition, and will determine if the most promising combinations of LRAs and CD8^+^ T-cells can eradicate the viral reservoir from natural patient-derived resting CD4^+^ T-cell reservoirs.

## Materials and Methods

### Ethics statement

HIV-infected individuals were recruited from the Maple Leaf Medical Clinic in Toronto, Canada through a protocol approved by the University of Toronto Institutional Review Board and from the Boston area (United States) under a protocol approved by the Institution Review Board at the Massachusetts General Hospital. Secondary use of the samples from Toronto was approved through the Massachusetts General Hospital Institutional Review Board. All subjects were adults, and gave written informed consent.

### T-cell cloning and maintenance

CD8^+^ T-cell responses in subjects were mapped by IFN-γ ELISPOT using 270 previously defined HIV optimal CD8^+^ epitopes restricted by common HLA alleles. For each response, PBMC were plated at 1x10^7^ cells/well in a 24-well plate and stimulated with 10 μg/ml of peptide for 3 hours. T-cells that had produced IFN-γ in response to this stimulation were enriched using the IFN-γ secretion detection and enrichment kit (Miltenyi Bioetc) following the manufacturer’s instructions. These cells were plated at a series of dilutions in 96-well plates with feeder medium (RPMI 1640 supplemented with 10% FBS and PenStrep [RPMI-10] with 1x10^6^ cells/ml 5,000 rad irradiated PBMC + 50 U/ml IL-2 + 0.1 μg/ml each of anti-CD3 (OKT3, ebioscience), anti-CD28 (CD28.2, ebioscience). One month later, colonies were selected from the lowest dilution plate with positive wells (<1/5 of wells positive) and screened for responsiveness to peptide by IFN-γ ELISPOT. Positive clones were expanded bi-weekly with feeder medium. Clone specificities were confirmed by degranulation assay (CD107a flow cytometry) on the day prior to recognition/elimination/eradication assays (see for ex. **Fig 3A**). Clones were washed extensively and resuspended in RPMI-10 media (no IL-2) prior to use in assays.

### Production of HIV stocks

Two methods were used to generate HIV stocks for experiments in this study. For the experiments depicted in **Figs 1 & 2**, stocks of the primary isolate HIV viruses 90TH_BK132, US1_GS004/7, and J3222 were obtained from the NIH AIDS reagent program. These viruses were amplified on purified (negatively selected) primary CD4^+^ T-cells that had been activated with 1 μg/ml each of anti-CD3 (OKT3, ebioscience), anti-CD28 (CD28.2, ebioscience), and 50 U/ml IL-2 (NIH AIDS reagent program). For the remainder of experiments, 293T cells were transfected with viral plasmids (JR-CSF, NL4-3, or LAI) using FuGENE HD (Promega) following the manufacturer’s instructions.

### HIV infections

#### Preparation of target cells

*Direct infection latency model*–total CD4^+^ T-cells were enriched from PBMC by negative selection (Easysep, Stemcell) following the manufacturer’s instructions, treated with 25 nM CCL19 (R&D Systems) for three hours, and then were infected without further manipulations (depletions of activated cells were performed after infection, immediately before use in downstream assays).

*‘Productively’ infected cells—*CD4^+^ T-cells were enriched as above and then activated for 48 hours with 1 μg/ml each of anti-CD3 (OKT3, ebioscience), anti-CD28 (CD28.2, ebioscience), and 50 U/ml IL-2 (NIH AIDS reagent program).

#### Infections

We used two different infection protocols in this study. In both cases, viral stocks were purified through a 20% sucrose cushion by centrifugation at maximal speed (20,800 x g) for 1 hour in a microcentrifuge at 4°C. Viral pellets were then resuspended in 1 ml of cold PBS and centrifuged as above. This wash step was repeated 3x and served to purify virus away from cytokines, particularly important for viral stocks that had been produced on activated primary CD4^+^ T-cells.

#### Method 1 –Magnetofection

In the experiments depicted in **Figs 1 & 2** we utilized a magnetofection protocol. Aiming for an MOI of ~1, we resuspended virus in 20 μl cold PBS and added 8 μl of magnetic ViroMag beads (OZ Biosciences) per million CD4^+^ T-cells. This was incubated at room temperature for 10 minutes and then mixed with target CD4^+^ T-cells at a concentration of 1x10^7^ cells/ml in RPMI-10 medium. This mixture was added to a 96-well flat bottom plate at 100 μl/well. The plate was then centrifuged at 800 x g for 5 minutes and placed on a magnetic plate (OZ Biosciences) for 1 hour at 37°C 5% CO_2_. The plate was then removed from the magnet, cells were washed 1x with RPMI-10 in 1.5 ml tubes, resuspended in 0.5 ml RPMI-10 in 24-well plates and returned to the incubator. Infections were monitored starting 18 hours later by surface staining with anti-CD4 APC (OKT4, Biolegend) and intracellularly with anti-HIV-Gag (Kc57, Beckman Coulter) following fixation/permeabilization using the BD cytofix/cytoperm system following the manufacturer’s instructions.

#### Method 2 –Spinoculation

In the remaining experiments we utilized a spinoculation method. Viral stocks were prepared in an identical manner but were not combined with ViroMag beads. Following addition of virus to CD4^+^ T-cells (1x10^6^ cells/well in 96-well flat bottom plates) plates were centrifuged at 1,200 x g for 90 minutes at 4°C. Cells were then washed 1x with RPMI-10 in 1.5 ml tubes, resuspended in 0.5 ml RPMI-10 in 24-well plates and returned to the incubator. Infections were monitored as above.

#### Generation of direct infection primary cell latency model

Direct-infection latency model target cells were prepared as above. Immediately prior to the initiation of assays, cells were stained with pooled PE conjugated antibodies directed against CD69, CD25, and HLA-DR (all from Biolegend, stained at 5 μl antibody in 100 μl 1% FBS PBS buffer). Cells were then washed, labeled with anti-PE microbeads (Miltenyi Bioetc) following the manufacturer’s instructions, and depleted of labeled cells using an AutoMACS system.

### Latency reversing agents

Suberoylanilide hydroxamic acid (SAHA) (Sigma-Aldrich), prostratin (Sigma-Aldrich), romidepsin (Selleckchem), panobinostat (Selleckchem), and hexamethylene bisacetamide (HMBA) (Sigma-Aldrich) were dissolved in hybrimax DMSO (Sigma-Aldrich) at the indicated concentrations. IL-7, IL-15, and IL-2 were purchased from R&D Systems and dissolved in sterile PBS. IL-15SA was generated by dissolving IL-15 and IL-15Rα-Fc (R&D Systems) in sterile PBS and combining these in equimolar ratios. Stocks of the above reagents were flash-frozen in single-use aliquots in EtOH dry-ice baths. ALT-803 was obtained from Altor Bioscience Corporation and stored at 4°C.

### Testing CD8^+^ T-cell recognition of latently-infected versus productively infected CD4^+^ T-cells (Fig 2A & 2B)

Latently-infected (direct-infection model) and productively-infected CD4^+^ T-cells were prepared as above. For the former, target cells were cultured with the indicated CD8^+^ T-cell clones in the presence of 1 μg/ml Brefeldin A (Sigma) and a PE-conjugated anti-CD107a antibody (Biolegend) immediately after depletion of activated cells. Note that in addition to facilitating CD107a staining, the Brefeldin A serves to prevent any MHC-I presentation of HIV antigens synthesized *de novo* during co-culture–thus the antigen presentation profile of the resting cells immediately after depletion of activated cells was queried. Productively infected cells were washed 3x prior to co-culture with CD8^+^ T-cells. For both sets of target cells, co-cultures were allowed to proceed for 16 hours. Cells were then surface stained with fluorochrome-conjugated antibodies to CD3, CD8, permeabilized (BD cytofix/cytoperm) and stained intracellularly with fluorochrome-conjugated anti-IFN-γ. Cells were fixed in 2% paraformaldehyde and analyzed on a FACSCalibur instrument (BD) and Flowjo software (TreeStar).

### Primary cell model latency reversal recognition assays

#### CD137 flow cytometry assay

Latently-infected CD4^+^ T-cells were prepared as above and then 72 hours later were co-cultured with the indicated CD8^+^ T-cell clones and candidate LRAs for 24 hours in RPMI-10 medium 1 μM each 3TC, AZT, and nevirapine without Brefeldin A. Cells were then stained with fluorochrome conjugated antibodies to CD3, CD8, CD4, and CD137 (4-1BB) all from Biolegend. Cells were fixed in 2% paraformaldehyde and analyzed on a FACSCalibur instrument (BD) and Flowjo software (TreeStar).

#### IFN-γ ELISA assay

*LRA wash-out variation* (**Fig 4**)- Latently-infected CD4^+^ T-cells were prepared as above and then 72 hours later added to 96-well round bottom plates at 100,000 cells/well in RPMI-10 medium with 1 μM each nevirapine and tenofovir. LRAs were added at the indicated concentrations and incubated for 72 hours. LRAs were then washed 3x, and medium was replaced with fresh RPMI-10 + nevirapine and tenofovir. CD8^+^ T-cell clones were washed 3x and added to these 96-well plates at 25,000 cells/well. Co-cultures were incubated for 16 hours and supernatants were harvested for IFN-γ ELISA.

*Continuous co-culture variation* (**Fig 3**)- As above, except cells were treated with LRAs for 72 hours and then CD8^+^ T-cell clones were added for an additional 72 hours, without a prior wash-step.

### Productively-infected cell killing assays

Primary CD4^+^ T-cells were enriched from PBMC by negative selection (Easysep, Stemcell Technologies) and then activated for 48 hours with 1 μg/ml each of anti-CD3 (OKT3, ebioscience) and anti-CD28 (CD28.2, ebioscience) in RPMI-10 supplemented with 50 U/ml IL-2. These cells were infected by a spinoculation method (see above). The following day, these target cells were co-cultured with effectors. Effectors were either: i) washed autologous CD8^+^ T-cell clones (taken at least 3 weeks post re-stimulation) at the indicated clone:target ratios (**Fig 7A–7C**), or autologous bulk CD8^+^ T-cells that had been isolated by negative selection (Easysep, Stemcell Technologies) (**Fig 7D**). For experiments with CD8^+^ T-cell clones, LRA treatments were peformed for a total of 60 hours. For the romidepsin treatment, cells were washed after 3 hours and medium was replaced with RPMI-10 + 50U/ml IL-2 for the remaining 57 hours. For the prostratin treatment cells were washed after 40 hours and given a 20 hour ‘rest’ in RPMI-10 + 50U/ml IL-2, for all other conditions LRAs were left in throughout the 60 hours and then washed repeatedly. For experiments with bulk CD8^+^ T-cells, ALT-803, IL-2, IL-7, and Pam3CSK4 LRAs were left in throughout a 16 hour co-culture period, whereas panobinostat, SAHA, and bryostatin, cells were added for 3 hours and then drugs were washed out for remaining co-culture period. In either case, bulk CD8^+^ T-cells were washed 3x prior to co-culture with target cells. Following 16 hours of co-culture, cells were stained with fluorochrome conjugated antibodies to CD3, CD4, and CD8 (all from Biolegend) as well as with blue viability dye (Life Technologies). Cells were then permeabilized (BD cytofix/cytoperm), then stained intracellularly with anti-HIV-Gag PE (Kc57, Beckman Coulter) and analyzed on a LSR-II instrument (BD) with FlowJo software (TreeStar). Levels of infection were assessed as %Gag^+^CD4^dim^ within the viable, CD3^+^CD8^-^ population.

### Natural reservoir latency reversal recognition assays

PBMC from ARV-treated HIV-infected subjects (HLA-A02^+^ and HLA-A02^-^) were depleted of CD8^+^ T-cells using Dynalbeads (Life Technologies). The remaining cells were stained with a cocktail of PE-conjugated antibodies against CD69, HLA-DR, and CD25 used at 25 μl antibody / 0.5 ml of 2% FBS PBS staining buffer for each 1 x 10^8^ cells (antibodies from Biolegend), for 15 minutes on ice. Cells were then washed and labeled with anti-PE microbeads (Miltenyi Biotec) using 75 μl beads in 300 μl of 2% FBS PBS per 1 x 10^8^ cells, on ice for 15 minutes. Cells were washed and re-suspended in 1 ml of 2% FBS PBS. An aliquot was stained with antibodies to CD3, CD4, and CD8 for flow cytometry analysis (pre-selection sample), and the remaining cells were separated by passing over MS columns (Miltenyi Biotec), following the manufacturer’s instructions. Post-selection samples were stained as above. The negative fraction of cells were stimulated with 0.71 nM ALT-803, or maintained as untreated controls for 72 hours in the presence of 1 μM each of tenofovir and emtricitabine in RPMI-10 medium. These target cells were washed, plated at 300,000 cells/well in a 96-well round bottom plate and then co-cultured with an HIV-Gag-SL9-specific CD8^+^ T-cell clone (20,000 cells/well) used 4 weeks post re-stimulation in the presence of 10 μg/ml of purified NA/LE anti-MHC-I (DX17, BD) or NA/LE IgG_1_ isotype control for 16 hours. IFN-γ in supernatants was quantified by ELISA (Biolegend).

### Assessing *ex vivo* activation of HIV transcription from natural reservoir

HIV-infected participants were selected based on sustained plasma viral load suppression (<50 copies/ml for >12 months), CD4 counts (>350 cells/μL), and absence of co-infection with hepatitis B or C virus. Clinical laboratory results were reconfirmed 2 weeks before leukapheresis or blood draw. Leukapheresis was conducted for 3–4 hours, and samples were processed within 2 hours after collection. The leukapheresis product was diluted 1:1 with PBS and layered over Ficoll for isolation of PBMCs. PBMCs were treated with red blood cell lysis buffer (eBioscience) and rested overnight (1 x 10^7^ cells/ml) in RPMI-10. To assess HIV activation, 15 million PBMCs were plated in 6-well plates in 5 ml of media, supplemented with antiretrovirals (ARVs) (100 nM elvitegravir and 100–300 nM efavirenz) for the entire duration of culture incubation. ALT-803 was added at 1 nM for 7 days. To measure HIV RNA levels, 1 ml of culture supernatant was analyzed by a robotic COBAS AmpliPrep/TaqMan system (Roche Diagnostics), which extracts total nucleic acid and quantifies HIV RNA in copies per milliliter using the HIV-1 Test, v2.0 kit (Roche Diagnostics).

### Primary CD4^+^ T cell HIV latency model

Total peripheral blood mononuclear cells (PBMCs) were obtained from healthy HIV-negative donors by leukapheresis (AllCells, Inc, Emeryville, CA). Naive CD4^+^ T cells were purified by negative selection using EasySep magnetic beads (StemCells, Inc) and cultured in RPMI with 10% fetal bovine serum (FBS), penicillin/streptomycin, 1% nonessential amino acids (Life Technologies), 1% sodium pyruvate (Life Technologies) and 495 nM beta-mercaptoethanol (Sigma Aldrich) in a 37°C, 5% CO_2_ incubator. Purified naive CD4^+^ T cells were activated by incubation with anti-CD3/CD28 magnetic Dynabeads (1 bead: 2 cells ratio, Life Technologies), 1 μg/ml anti-IL-4 (R&D Systems), 2 μg/ml anti-IL-12p70 antibodies (R&D Systems), and 10 ng/ml TGF-β (R&D Systems) for 3 days [61,62]. Following the removal of anti-CD3/CD28 beads and antibodies, cells were maintained in 30 U/ml IL-2 (Life Technologies) for 2 days. Cells were then infected with NL4.3-Luc in the presence of 50 μg/ml DEAE for 3 hours. Cells were maintained in the continued presence of 30 U/ml IL-2 throughout the infection and subsequent rest period with culture medium with fresh IL-2 replaced every 2–3 days. Seven days post-infection, 20 μl of latently infected cells were dispensed into 384 well plates using a MicroFlo dispenser (Biotek Instruments) at 10,000 cells/well containing 100 nl of compound solutions delivered by the Echo acoustic-based liquid dispenser (Labcyte). After a 48-hour incubation, 16 μl/well BriteGlo (Promega) was added and luminescence measured using the Envision plate reader (Perkin Elmer).

### Assessing toxicity of LRAs in post-activation primary cell model

Compound-associated cytotoxicity was determined in latently infected cells in parallel with the virus reactivation assay. Cells were incubated with compounds for 48 hours at 37°C, and cell viability was determined using Cell Titer Glo reagent (Promega).

### Quantitative PCR

HIV viral RNA was quantified by real-time RT-PCR using a probe based method. Reactions were performed with AgPath-ID one-step RT-PCR mastermix (Life Technologies) following the manufacturer’s instructions with the following primers/probes: HIV-pol–fprimer GCACTTTAAATTTTCCCATTAGTCCTA, rprimer CAAATTTCTACTAATGCTTTTATTTTTTC, probe FAM-AAGCCAGGAATGGATGGCC-MGBNFQ. Absolute quantifications were established by comparison to a standard curve generated with linearized pUC57 plasmid standard with the insert GCACTTTAAATTTTCCCATTAGTCCTATTGAAACTGTACCAGTAAAATTAAAGCCAGGAATGGATGGCCCAAAAGTCAAACAATGGCCATTGACAGAAGAAAAAATAAAAGCATTAGTAGAAATTTG. Reactions were performed and read by a Roche LightCycler 96 well system.

### Droplet digital PCR

Genomic DNA was extracted using the Gentra Puregene kit (Gentra) following the manufacturer’s instructions. For each sample, 5 μg DNA was digested with the BsaJI enzyme (NEB) in 1x NEB smartcut buffer. DNA was heated to 95°C for 10 minutes then chilled on ice before addition of the BsaJI enzyme. Digestions were performed for 1 hour at 60°C. DNA was then purified using the GeneJET PCR purification kit (Life Technologies). PCR reactions comprised 1x ddPCR Supermix for Probes (Bio-Rad), 18 μM each of primers, 5 μM probe, 10 units/reaction BSAJI enzyme, and 0.2 μg DNA. Primers/Probes were: RPP30 –fprimer GATTTGGACCTGCGAGCG, rprimer GCGGCTGTCTCCACAAGT, probe VIC-CTGAACTGAAGGCTCT-MGBNFQ; HIV-gag–fprimer TACTGACGCTCTCGCACC, rprimer TCTCGACGCAGGACTCG, probe FAM-CTCTCTCCTTCTAGCCTC-MGBNFQ. Droplets were prepared using the QX100 Droplet Generator (Bio-Rad) following the manufacturer’s instructions. Sealed plates were cycled using the following program: 95°C for 10 min; 40 cycles of 94°C for 30 s, 60°C for 1 min; and 98°C for 10 min. Reactions were analyzed using the QX100 Droplet Reader and number of template molecule per μl of starting material was estimated using the Quantalife ddPCR software.

### Statistical analysis

Statistical analyses were performed using Prism software (Graphpad). The statistical tests used to calculate p values are indicated in the corresponding figure legends.

## References

[1] Rueda S, Law S, Rourke SB (2014) Psychosocial, mental health, and behavioral issues of aging with HIV. Curr Opin HIV AIDS 9: 325–331. 10.1097/COH.0000000000000071 24824890

[2] Arseniou S, Arvaniti A, Samakouri M (2014) HIV infection and depression. Psychiatry Clin Neurosci 68: 96–109. 10.1111/pcn.12097 24552630

[3] Katz IT, Ryu AE, Onuegbu AG, Psaros C, Weiser SD, et al (2013) Impact of HIV-related stigma on treatment adherence: systematic review and meta-synthesis. J Int AIDS Soc 16: 18640 10.7448/IAS.16.3.18640 24242258 PMC3833107

[4] Chun TW, Finzi D, Margolick J, Chadwick K, Schwartz D, et al (1995) In vivo fate of HIV-1-infected T cells: quantitative analysis of the transition to stable latency. Nat Med 1: 1284–1290. 7489410 10.1038/nm1295-1284

[5] Chun TW, Stuyver L, Mizell SB, Ehler LA, Mican JA, et al (1997) Presence of an inducible HIV-1 latent reservoir during highly active antiretroviral therapy. Proc Natl Acad Sci U S A 94: 13193–13197. 9371822 10.1073/pnas.94.24.13193 PMC24285

[6] Finzi D, Hermankova M, Pierson T, Carruth LM, Buck C, et al (1997) Identification of a reservoir for HIV-1 in patients on highly active antiretroviral therapy. Science 278: 1295–1300. 9360927 10.1126/science.278.5341.1295

[7] Wong JK, Hezareh M, Gunthard HF, Havlir DV, Ignacio CC, et al (1997) Recovery of replication-competent HIV despite prolonged suppression of plasma viremia. Science 278: 1291–1295. 9360926 10.1126/science.278.5341.1291

[8] Finzi D, Blankson J, Siliciano JD, Margolick JB, Chadwick K, et al (1999) Latent infection of CD4+ T cells provides a mechanism for lifelong persistence of HIV-1, even in patients on effective combination therapy. Nat Med 5: 512–517. 10229227 10.1038/8394

[9] Heslop HE, Slobod KS, Pule MA, Hale GA, Rousseau A, et al (2010) Long-term outcome of EBV-specific T-cell infusions to prevent or treat EBV-related lymphoproliferative disease in transplant recipients. Blood 115: 925–935. 10.1182/blood-2009-08-239186 19880495 PMC2817637

[10] Deeks SG (2012) HIV: Shock and kill. Nature 487: 439–440. 10.1038/487439a 22836995

[11] Bullen CK, Laird GM, Durand CM, Siliciano JD, Siliciano RF (2014) New ex vivo approaches distinguish effective and ineffective single agents for reversing HIV-1 latency in vivo. Nat Med 20: 425–429. 10.1038/nm.3489 24658076 PMC3981911

[12] Rasmussen TA, Schmeltz Sogaard O, Brinkmann C, Wightman F, Lewin SR, et al (2013) Comparison of HDAC inhibitors in clinical development: effect on HIV production in latently infected cells and T-cell activation. Hum Vaccin Immunother 9: 993–1001. 10.4161/hv.23800 23370291 PMC3899169

[13] Contreras X, Schweneker M, Chen CS, McCune JM, Deeks SG, et al (2009) Suberoylanilide hydroxamic acid reactivates HIV from latently infected cells. J Biol Chem 284: 6782–6789. 10.1074/jbc.M807898200 19136668 PMC2652322

[14] Archin NM, Espeseth A, Parker D, Cheema M, Hazuda D, et al (2009) Expression of latent HIV induced by the potent HDAC inhibitor suberoylanilide hydroxamic acid. AIDS Res Hum Retroviruses 25: 207–212. 10.1089/aid.2008.0191 19239360 PMC2853863

[15] Mohammadi P, di Iulio J, Munoz M, Martinez R, Bartha I, et al (2014) Dynamics of HIV latency and reactivation in a primary CD4+ T cell model. PLoS Pathog 10: e1004156 10.1371/journal.ppat.1004156 24875931 PMC4038609

[16] Saleh S, Wightman F, Ramanayake S, Alexander M, Kumar N, et al (2011) Expression and reactivation of HIV in a chemokine induced model of HIV latency in primary resting CD4+ T cells. Retrovirology 8: 80 10.1186/1742-4690-8-80 21992606 PMC3215964

[17] Wei DG, Chiang V, Fyne E, Balakrishnan M, Barnes T, et al (2014) Histone deacetylase inhibitor romidepsin induces HIV expression in CD4 T cells from patients on suppressive antiretroviral therapy at concentrations achieved by clinical dosing. PLoS Pathog 10: e1004071 10.1371/journal.ppat.1004071 24722454 PMC3983056

[18] Blazkova J, Chun TW, Belay BW, Murray D, Justement JS, et al (2012) Effect of histone deacetylase inhibitors on HIV production in latently infected, resting CD4(+) T cells from infected individuals receiving effective antiretroviral therapy. J Infect Dis 206: 765–769. 10.1093/infdis/jis412 22732922 PMC3491743

[19] Laird GM, Bullen CK, Rosenbloom DI, Martin AR, Hill AL, et al (2015) Ex vivo analysis identifies effective HIV-1 latency-reversing drug combinations. J Clin Invest 125: 1901–1912. 10.1172/JCI80142 25822022 PMC4463209

[20] Cillo AR, Sobolewski MD, Bosch RJ, Fyne E, Piatak M Jr., et al (2014) Quantification of HIV-1 latency reversal in resting CD4+ T cells from patients on suppressive antiretroviral therapy. Proc Natl Acad Sci U S A 111: 7078–7083. 10.1073/pnas.1402873111 24706775 PMC4024870

[21] Elliott JH, Wightman F, Solomon A, Ghneim K, Ahlers J, et al (2014) Activation of HIV transcription with short-course vorinostat in HIV-infected patients on suppressive antiretroviral therapy. PLoS Pathog 10: e1004473 10.1371/journal.ppat.1004473 25393648 PMC4231123

[22] Archin NM, Liberty AL, Kashuba AD, Choudhary SK, Kuruc JD, et al (2012) Administration of vorinostat disrupts HIV-1 latency in patients on antiretroviral therapy. Nature 487: 482–485. 10.1038/nature11286 22837004 PMC3704185

[23] Irvine DJ, Purbhoo MA, Krogsgaard M, Davis MM (2002) Direct observation of ligand recognition by T cells. Nature 419: 845–849. 12397360 10.1038/nature01076

[24] Lassen KG, Bailey JR, Siliciano RF (2004) Analysis of human immunodeficiency virus type 1 transcriptional elongation in resting CD4+ T cells in vivo. J Virol 78: 9105–9114. 15308706 10.1128/JVI.78.17.9105-9114.2004 PMC506937

[25] Graf EH, Pace MJ, Peterson BA, Lynch LJ, Chukwulebe SB, et al (2013) Gag-positive reservoir cells are susceptible to HIV-specific cytotoxic T lymphocyte mediated clearance in vitro and can be detected in vivo [corrected]. PLoS One 8: e71879 10.1371/journal.pone.0071879 23951263 PMC3737195

[26] Pace MJ, Graf EH, Agosto LM, Mexas AM, Male F, et al (2012) Directly infected resting CD4+T cells can produce HIV Gag without spreading infection in a model of HIV latency. PLoS Pathog 8: e1002818 10.1371/journal.ppat.1002818 22911005 PMC3406090

[27] Jones RB, Garrison KE, Mujib S, Mihajlovic V, Aidarus N, et al (2012) HERV-K-specific T cells eliminate diverse HIV-1/2 and SIV primary isolates. J Clin Invest 122: 4473–4489. 10.1172/JCI64560 23143309 PMC3533554

[28] Garrison KE, Jones RB, Meiklejohn DA, Anwar N, Ndhlovu LC, et al (2007) T cell responses to human endogenous retroviruses in HIV-1 infection. PLoS Pathog 3: e165 17997601 10.1371/journal.ppat.0030165 PMC2065876

[29] Sahu GK, Lee K, Ji J, Braciale V, Baron S, et al (2006) A novel in vitro system to generate and study latently HIV-infected long-lived normal CD4+ T-lymphocytes. Virology 355: 127–137. 16919704 10.1016/j.virol.2006.07.020

[30] Wolfl M, Kuball J, Ho WY, Nguyen H, Manley TJ, et al (2007) Activation-induced expression of CD137 permits detection, isolation, and expansion of the full repertoire of CD8+ T cells responding to antigen without requiring knowledge of epitope specificities. Blood 110: 201–210. 17371945 10.1182/blood-2006-11-056168 PMC1896114

[31] Jones RB, O'Connor R, Mueller S, Foley M, Szeto GL, et al (2014) Histone deacetylase inhibitors impair the elimination of HIV-infected cells by cytotoxic T-lymphocytes. PLoS Pathog 10: e1004287 10.1371/journal.ppat.1004287 25122219 PMC4133386

[32] Lehrman G, Ylisastigui L, Bosch RJ, Margolis DM (2004) Interleukin-7 induces HIV type 1 outgrowth from peripheral resting CD4+ T cells. J Acquir Immune Defic Syndr 36: 1103–1104. 15247565 10.1097/00126334-200408150-00015

[33] Wang FX, Xu Y, Sullivan J, Souder E, Argyris EG, et al (2005) IL-7 is a potent and proviral strain-specific inducer of latent HIV-1 cellular reservoirs of infected individuals on virally suppressive HAART. J Clin Invest 115: 128–137. 15630452 10.1172/JCI22574 PMC539197

[34] Novis CL, Archin NM, Buzon MJ, Verdin E, Round JL, et al (2013) Reactivation of latent HIV-1 in central memory CD4(+) T cells through TLR-1/2 stimulation. Retrovirology 10: 119 10.1186/1742-4690-10-119 24156240 PMC3826617

[35] Klichko V, Archin N, Kaur R, Lehrman G, Margolis D (2006) Hexamethylbisacetamide remodels the human immunodeficiency virus type 1 (HIV-1) promoter and induces Tat-independent HIV-1 expression but blunts cell activation. J Virol 80: 4570–4579. 16611917 10.1128/JVI.80.9.4570-4579.2006 PMC1472000

[36] Choudhary SK, Archin NM, Margolis DM (2008) Hexamethylbisacetamide and disruption of human immunodeficiency virus type 1 latency in CD4(+) T cells. J Infect Dis 197: 1162–1170. 10.1086/529525 18419522

[37] Shan L, Deng K, Shroff NS, Durand CM, Rabi SA, et al (2012) Stimulation of HIV-1-specific cytolytic T lymphocytes facilitates elimination of latent viral reservoir after virus reactivation. Immunity 36: 491–501. 10.1016/j.immuni.2012.01.014 22406268 PMC3501645

[38] Ho YC, Shan L, Hosmane NN, Wang J, Laskey SB, et al (2013) Replication-competent noninduced proviruses in the latent reservoir increase barrier to HIV-1 cure. Cell 155: 540–551. 10.1016/j.cell.2013.09.020 24243014 PMC3896327

[39] Pollack R (2015) Patient-derived defective HIV-1 proviruses containing large internal deletions can be transcribed and translated. Towards an HIV Cure Symposium. Vancouver.

[40] Jayakumar A, Castilho TM, Park E, Goldsmith-Pestana K, Blackwell JM, et al (2011) TLR1/2 activation during heterologous prime-boost vaccination (DNA-MVA) enhances CD8+ T Cell responses providing protection against Leishmania (Viannia). PLoS Negl Trop Dis 5: e1204 10.1371/journal.pntd.0001204 21695103 PMC3114751

[41] Asprodites N, Zheng L, Geng D, Velasco-Gonzalez C, Sanchez-Perez L, et al (2008) Engagement of Toll-like receptor-2 on cytotoxic T-lymphocytes occurs in vivo and augments antitumor activity. FASEB J 22: 3628–3637. 10.1096/fj.08-108274 18587008 PMC2537425

[42] Hernandez-Ruiz J, Salaiza-Suazo N, Carrada G, Escoto S, Ruiz-Remigio A, et al (2010) CD8 cells of patients with diffuse cutaneous leishmaniasis display functional exhaustion: the latter is reversed, in vitro, by TLR2 agonists. PLoS Negl Trop Dis 4: e871 10.1371/journal.pntd.0000871 21072232 PMC2970528

[43] Ahmad A, Ahmad R, Iannello A, Toma E, Morisset R, et al (2005) IL-15 and HIV infection: lessons for immunotherapy and vaccination. Curr HIV Res 3: 261–270. 16022657 10.2174/1570162054368093

[44] Chehimi J, Marshall JD, Salvucci O, Frank I, Chehimi S, et al (1997) IL-15 enhances immune functions during HIV infection. J Immunol 158: 5978–5987. 9190952

[45] D'Offizi G, Gioia C, Corpolongo A, Martini F, Paganelli R, et al (2007) An IL-15 dependent CD8 T cell response to selected HIV epitopes is related to viral control in early-treated HIV-infected subjects. Int J Immunopathol Pharmacol 20: 473–485. 17880761 10.1177/039463200702000306

[46] Gomes-Giacoia E, Miyake M, Goodison S, Sriharan A, Zhang G, et al (2014) Intravesical ALT-803 and BCG treatment reduces tumor burden in a carcinogen induced bladder cancer rat model; a role for cytokine production and NK cell expansion. PLoS One 9: e96705 10.1371/journal.pone.0096705 24896845 PMC4045574

[47] Mueller YM, Bojczuk PM, Halstead ES, Kim AH, Witek J, et al (2003) IL-15 enhances survival and function of HIV-specific CD8+ T cells. Blood 101: 1024–1029. 12393488 10.1182/blood-2002-07-1957

[48] Stephan MT, Moon JJ, Um SH, Bershteyn A, Irvine DJ (2010) Therapeutic cell engineering with surface-conjugated synthetic nanoparticles. Nat Med 16: 1035–1041. 10.1038/nm.2198 20711198 PMC2935928

[49] White L, Krishnan S, Strbo N, Liu H, Kolber MA, et al (2007) Differential effects of IL-21 and IL-15 on perforin expression, lysosomal degranulation, and proliferation in CD8 T cells of patients with human immunodeficiency virus-1 (HIV). Blood 109: 3873–3880. 17192392 10.1182/blood-2006-09-045278 PMC1874576

[50] Wong HC, Jeng EK, Rhode PR (2013) The IL-15-based superagonist ALT-803 promotes the antigen-independent conversion of memory CD8 T cells into innate-like effector cells with antitumor activity. Oncoimmunology 2: e26442 24404427 10.4161/onci.26442 PMC3881336

[51] Xin KQ, Hamajima K, Sasaki S, Tsuji T, Watabe S, et al (1999) IL-15 expression plasmid enhances cell-mediated immunity induced by an HIV-1 DNA vaccine. Vaccine 17: 858–866. 10067692 10.1016/s0264-410x(98)00271-0

[52] Xu W, Jones M, Liu B, Zhu X, Johnson CB, et al (2013) Efficacy and mechanism-of-action of a novel superagonist interleukin-15: interleukin-15 receptor alphaSu/Fc fusion complex in syngeneic murine models of multiple myeloma. Cancer Res 73: 3075–3086. 10.1158/0008-5472.CAN-12-2357 23644531 PMC3914673

[53] Rosenberg SA (2014) IL-2: the first effective immunotherapy for human cancer. J Immunol 192: 5451–5458. 10.4049/jimmunol.1490019 24907378 PMC6293462

[54] Deng K, Pertea M, Rongvaux A, Wang L, Durand CM, et al (2015) Broad CTL response is required to clear latent HIV-1 due to dominance of escape mutations. Nature 517: 381–385. 10.1038/nature14053 25561180 PMC4406054

[55] Lee SM, Joo YD, Seo SK (2009) Expression and Function of TLR2 on CD4 Versus CD8 T Cells. Immune Netw 9: 127–132. 10.4110/in.2009.9.4.127 20157599 PMC2816945

[56] Sung JA, Lam S, Garrido C, Archin N, Rooney CM, et al (2015) Expanded cytotoxic T-cell lymphocytes target the latent HIV reservoir. J Infect Dis 212: 258–263. 10.1093/infdis/jiv022 25589335 PMC4490234

[57] Zhu X, Marcus WD, Xu W, Lee HI, Han K, et al (2009) Novel human interleukin-15 agonists. J Immunol 183: 3598–3607. 10.4049/jimmunol.0901244 19710453 PMC2814526

[58] Han KP, Zhu X, Liu B, Jeng E, Kong L, et al (2011) IL-15:IL-15 receptor alpha superagonist complex: high-level co-expression in recombinant mammalian cells, purification and characterization. Cytokine 56: 804–810. 10.1016/j.cyto.2011.09.028 22019703 PMC3221918

[59] Mathios D, Park CK, Marcus WD, Alter S, Rhode PR, et al (2015) Therapeutic administration of IL-15 superagonist complex ALT-803 leads to long-term survival and durable antitumor immune response in a murine glioblastoma model. Int J Cancer. 10.1002/ijc.29686 PMC4696021 26174883

[60] Seay K, Church C, Zheng JH, Deneroff K, Ochsenbauer C, et al (2015) In Vivo Activation of Human NK Cells by Treatment with an Interleukin-15 Superagonist Potently Inhibits Acute In Vivo HIV-1 Infection in Humanized Mice. J Virol 89: 6264–6274. 10.1128/JVI.00563-15 25833053 PMC4474292

[61] Bosque A, Famiglietti M, Weyrich AS, Goulston C, Planelles V (2011) Homeostatic proliferation fails to efficiently reactivate HIV-1 latently infected central memory CD4+ T cells. PLoS Pathog 7: e1002288 10.1371/journal.ppat.1002288 21998586 PMC3188522

[62] Messi M, Giacchetto I, Nagata K, Lanzavecchia A, Natoli G, et al (2003) Memory and flexibility of cytokine gene expression as separable properties of human T(H)1 and T(H)2 lymphocytes. Nat Immunol 4: 78–86. 12447360 10.1038/ni872

